# NeuroLab 2.0: An Alternative Storyline Design Approach for Translating a Research-Based Summer Experience into an Advanced STEM+M Curriculum Unit that Supports Three-Dimensional Teaching and Learning in the Classroom

**DOI:** 10.15695/jstem/v7i1.03

**Published:** 2024-03-06

**Authors:** Linda Santschi, Kristin M. Bass, Ralph Imondi

**Affiliations:** 1 Coastal Marine Biolabs Integrative Biosciences Program, Ventura Harbor, CA; 2 Rockman et al Cooperative, Berkeley, CA

**Keywords:** NeuroLab, Student Collaboration, Storylines, Scientific Modeling, Science Practices, NGSS, STEM+M, STEMM, High School Students, Precollege Students, Developmental Neuroscience, Clinical Neuroscience, Neurology, Big Data, Data Literacy, Researcher-Educator Collaboration, Interdisciplinary, Mirror Movement Disorder

## Abstract

In this case study, we describe an alternative storyline design approach that we adopted to translate an informal, out-of-school summer science experience with a strong emphasis on developmental neuroscience and data literacy into a more inclusive, replicable, and scalable experience for formal high school science instruction. Combining elements of problem- and project-based learning, a storyline is a curriculum model that engages students in the application of investigative science and engineering practices to incrementally build conceptual models that explain an observable (anchoring) phenomenon. Published reports on the storyline design process describe procedures and tools that are well suited to the creation of novel instructional units. However, these design methods are difficult to apply to projects aimed at translating pre-existing science experiences and resources into classroom storyline units. In this descriptive case study, we discuss a series of alternative design procedures that we utilized to achieve this adaptation. Our overarching project goal was to create the resources necessary to engage high school students in the construction of a multidimensional explanatory model for an unusual movement disorder that assimilates converging lines of behavioral, neuroanatomical, neurophysiological, molecular genetic, developmental, and cellular data. The methods described in this case study establish a design template for other biomedical scientists who are interested in adopting a storyline approach to bring aspects of their work or educational projects into science classrooms and into closer alignment with a new vision for science teaching and learning articulated in the National Research Council’s *A Framework for K-12 Science Education* and the *Next Generation Science Standards*.

## INTRODUCTION

This case study draws upon our work on two neuroscience-focused research education projects funded under the Science Education Partnership Award (SEPA) program of the National Institute of General Medical Sciences of the National Institutes of Health. Our goal is to provide practical guidance for scientists and curriculum designers who seek to adapt pre-existing learning experiences organized around specific biomedical phenomena into science, technology, engineering, mathematics, and medicine (STEM+M) storyline units that support student epistemic agency and incremental model building. To provide the appropriate context for our design work, we begin the case study with a brief description of the features and limitations of NeuroLab 1.0, an *informal*, out-of-school summer learning experience that we launched in 2014. We then introduce the collaborative storyline approach that we adopted to address these limitations and translate key aspects of the NeuroLab 1.0 informal science experience (ISE) into a novel instructional unit for science teachers to implement in *formal* classroom learning environments (the primary goal of NeuroLab 2.0 and the focus of this report). The specific steps of our storyline design process—which deviate from published storyline design sequences in a number of important ways (Penuel et al., 2022a,b)—are described in the sections that follow. We conclude our case report with several recommendations that emerged through our collaborative design effort.

In a previous report (Imondi et al., 2019), we described our work on NeuroLab 1.0, an informal ISE for upper-level high school students that unfolded in our biosciences laboratory under the direct supervision of neuroscientist instructor-mentors (NeuroLab 1.0 ISE). Modeled around course-based undergraduate research experiences (Auchincloss et al., 2014), this immersive out-of-school program recruited small cohorts of predominantly female students to participate in ten-day residential research experiences that bridged comparative functional genomics and developmental neuroscience. The hands-on component of the NeuroLab 1.0 ISE provided novel opportunities for students to screen a library of evolutionarily conserved regulatory elements (putative gene switches identified by the Lawrence Berkeley National Laboratory) for their ability to drive reporter gene expression in specific populations of embryonic spinal cord neurons (Visel et al., 2007, 2009; Imondi et al., 2019; Hadas et al., 2014). Student work was framed within the context of a logic model that emphasized the potential value of these genomic tools for filling gaps in our current understanding of nervous system assembly and for developing novel and highly targeted gene-based treatments for spinal cord disease and trauma affecting human movement. To help underscore the biomedical relevance of their hands-on work and reveal gaps in our current understanding of nervous system assembly, students examined and interpreted well-established models of axon pathfinding, the process by which the wire-like axons extending from embryonic neurons locate target cells during nervous system assembly (Comer et al., 2019; Araújo and Tear, 2003). Particular emphasis was placed on the molecular navigational systems that guide the growth cone—a motile and highly dynamic structure at the terminus of outgrowing axons—across the midline and toward distant target cells involved in coordinating movement between the two sides of the body (Dickson and Zou, 2010; Howard et al., 2019; Evans and Bashaw, 2010).

Despite its success in stimulating student interest and achieving targeted outcomes (e.g., positive changes in attitudes toward science and increased collaborative abilities, self-efficacy for conducting science research, and persistence on challenging tasks; Imondi et al., 2019), the NeuroLab 1.0 ISE engaged limited numbers of high-performing students, most of whom had already developed an interest in science and medicine. This limitation motivated our team to consider strategies for bringing key elements of this informal, out-of-school experience into high school classrooms to engage students with more mixed interests and academic performance histories, and to help teachers meet new instructional challenges imposed by the Next Generation Science Standards (NGSS; NGSS Lead States, 2013). Our immediate goal was to create the curricular resources necessary to support a more inclusive and scalable classroom experience that maintains the research focus, authenticity, interdisciplinary scope, and collaborative nature of the NeuroLab ISE while conforming, inasmuch as possible, to an ambitious new vision of science teaching and learning articulated in *A Framework for K-12 Science Education* (National Research Council, 2012) and the NGSS it guided. Based on these considerations and a long-standing programmatic goal to emphasize the critical role of models in driving biomedical inquiry, our focus turned to a storyline design approach.

As discussed elsewhere (Penuel et al., 2022a), the storyline approach seeks to create a collaborative environment for students to build knowledge collectively and incrementally through engagement in NGSS science and engineering practices (i.e., asking questions, developing and using models, planning and carrying out investigations, analyzing and interpreting data, using mathematics and computational thinking, constructing explanations, engaging in argument from evidence, obtaining, evaluating, and communicating information; NGSS Lead States, 2013). According to this approach, units are organized around an observable (anchoring) phenomenon that students are tasked with explaining through the application of these investigative practices. Motivation to pursue this central work task and a shared mission is established at the beginning of a storyline unit through the *anchoring phenomenon routine*, one of several instructional strategies that teachers utilize during storyline enactment (Penuel et al., 2022b). As its name implies, the *anchoring phenomenon routine* introduces students to the anchoring phenomenon and invites them to formulate questions based on their observations of the phenomenon, prior knowledge of (and/or experience with) related phenomena, and their early attempts to construct an explanatory model for the phenomenon. During this opening segment of the storyline, students and their teacher prioritize a series of questions to pursue through subsequent investigation, thereby defining the unit goals as a group. As students pursue investigations motivated by their own questions, they assimilate what they learned into interim explanatory models that are subject to classroom discussion and revision. The motivation to revise an emerging model and pursue further investigations is derived from the students’ own recognition that their construct contains gaps or deficiencies that diminish its explanatory power.

Our efforts to translate the NeuroLab 1.0 ISE into a storyline unit that supports student epistemic agency unfolded over three interrelated project phases: 1) the co-design of a storyline lesson sequence involving a small group of scientists and educators; 2) the design and implementation of a structured professional development program aimed at preparing high school science teachers to conduct implementation trials of the emerging storyline unit in an upper-level (11th and 12th grade) high school life science course (to provide actionable information on storyline design); and 3) teacher enactment of the storyline unit in the classroom (to provide preliminary efficacy data on targeted program outcomes and guidance on storyline revisions that enhance its ability to support student epistemic agency and coherence from the students’ perspective; Penuel et al., 2022a,b). In this descriptive case study, we describe work associated with the first phase of the project. Particular emphasis is placed on the approach used by our scientist-teacher partnership team to design a storyline framework aimed at engaging students in the creation of an explanatory model for an unusual and heritable movement disorder with strong conceptual linkages to axon pathfinding, a major emphasis of the NeuroLab 1.0 ISE. From a practical standpoint, the approach that we adopted was intended to culminate in the creation of innovative curriculum resources and strategies that teachers can bring into the high school science classroom to capture the most salient and successful aspects of the NeuroLab 1.0 ISE.

## METHODS

### Co-Design Team.

The storyline design team initially consisted of two Ph.D.-level neuroscientists with extensive research experience and technical expertise in the areas of neurophysiology and developmental neurobiology (project leaders), and five experienced high school science teachers (co-designers), four of whom are long-standing participants in a citizen science program hosted by our lab (Santschi et al., 2013; Henter et al., 2016). Table 1 presents a timeline of co-design segments and the primary activities associated with each segment. Prior to storyline design workshops, teachers attended an initial five-day summer institute (Table 1, row 1) that was primarily intended to: 1) clarify the goals of the NeuroLab 2.0 project and their alignment with the overarching goals of the SEPA program; 2) establish the roles and responsibilities of participants, project staff, and our collaborators; and 3) deepen teachers’ mastery of life science ideas and concepts encompassed by the NeuroLab 1.0 ISE experience that forms the foundation of the current translational effort. This experience was followed by a series of 12 one-day unit co-design workshops (Table 1, row 2), followed by a five-day post-workshop summer institute and 2 one-day concluding workshops (Table 1, rows 3 and 4).

Before storyline design workshops commenced, we created and administered a 17-item instrument to assess teachers’ incoming level of NGSS understanding and experience. As indicated in Table 1, building group knowledge around the storyline approach to unit design and its ties to the NRC Framework and the NGSS was an important component of the co-design process that was intended to build upon teachers’ incoming experience and level of understanding (as assessed by the information captured through the survey).

Fifteen additional teachers (early adopters) representing nine states (CA, FL, KS, MO, NC, NH, OR, RI, and TX) were recruited to conduct initial implementation trials of the storyline unit after attending nine-day professional development institutes hosted at our lab. The characteristics of these institutes and their direct impacts on teachers will be described in a separate publication along with a description of the resources developed to support classroom enactment of the storyline experience (which include physical artifacts generated in connection with the design process outlined in this report).

### Protection of Human Subjects.

The work described in this case study constitutes part of a larger research study that was reviewed and approved by Solutions IRB (Yarnell, AZ) prior to project launch (Federalwide Assurance [FWA] #: FWA00021831). In accordance with our approved protocol, all student work collected by co-designers, which included questions and explanatory models, were de-identified by teachers prior to their distribution to group members for analysis and discussion.

### Co-Design Process.

Our goal to adapt a prior ISE experience for the high school classroom established a markedly different starting point for storyline design than other approaches, which often begin with a consideration of targeted standards and involve a sequence of activities for development teams to brainstorm phenomena—and elaborate explanations (models) of phenomena—to evaluate their potential to address NGSS elements (see Penuel et al., 2022a and references therein). In this section, we describe an alternative design sequence adopted by our team, which was guided by our overall project goals and a desire to link the developmental neuroscience ideas, concepts, and models highlighted in the NeuroLab 1.0 ISE with the current storyline effort. The major steps comprising our co-design process are summarized in Table 2.

#### 1. Selection of an anchoring phenomenon with conceptual ties to the NeuroLab 1.0 ISE.

After reaching consensus on a storyline approach to unit development, we focused our efforts on the identification of an anchoring phenomenon with strong linkages to axon pathfinding, a central conceptual focus of the NeuroLab 1.0 ISE upon which the present project is based. As noted above, axon pathfinding (or axon guidance) refers to the dynamic process by which neurons find—and ultimately form functional connections with—target neurons during nervous system development.

The NeuroLab 1.0 ISE engaged students in the analysis and interpretation of axon guidance models that were constructed over the last several decades through the systematic study of model organisms and systems (e.g., the embryonic mouse spinal cord and hindbrain, the fruit fly and nematode worm ventral nerve cord, etc.). During these experiences, particular emphasis was placed on the prevailing model of *midline guidance*, which synthesizes an extensive body of evidence about the molecular navigational systems that embryonic neurons use to project their axons across the midline en route to contralateral target cells involved in coordinating movement between two sides of the body axis (Dickson and Zou, 2010; Howard et al., 2019; Evans and Bashaw, 2010). Observable behavioral phenotypes that arise in model vertebrate organisms (e.g., mouse and zebrafish; Jain et al., 2014, Finger et al., 2002, Bernhardt et al., 2012) as a result of midline pathfinding errors represented obvious candidates for an anchoring phenomenon with strong conceptual ties to the NeuroLab 1.0 ISE. However, group members uniformly agreed that organizing the NeuroLab 2.0 storyline around a human movement disorder that is linked to errors in midline pathfinding was more likely to capture and maintain the interest of their students and motivate them to figure out life science ideas and concepts encompassed by the NGSS.

Owing largely to lagging advances in neuroimaging technologies, a surprisingly small number of human disorders have been directly linked to axon guidance defects or genes implicated in this developmental process (Cox et al., 2012; Nugent et al., 2012; Van Battum et al., 2015; Bernhardt et al., 2013). Among the disorders identified to date, we focused our attention on congenital mirror movement disorder (CMM; OMIM# 157600, 618264) based on the following considerations:

- Affected individuals exhibit an unusual and readily discernible behavioral phenotype affecting movement;
- The behavioral phenotype exhibits superficial similarities to other movement disorders and syndromes that students are likely to have encountered in their everyday lives, regardless of their race, gender, or first language;
- Movement defects are clearly depicted in open-access videos in which affected individuals perform motor tasks in clinical settings (a compelling vehicle to launch the storyline unit);
- The disorder is linked to genes and interacting proteins with long-established roles in midline pathfinding in model organisms (a central conceptual focus of the NeuroLab 1.0 ISE that was specifically targeted for carryover to NeuroLab 2.0 in accordance with project goals);
- Movement abnormalities are linked to reasonably unambiguous and readily interpretable anatomical defects in the central nervous system that share qualitative similarities to defects observed in simpler model organisms; and
- A wealth of evidence to support incremental model building can be adapted from the scientific literature or accessed from authoritative databases that students can navigate (with guidance) during the storyline trajectory.

Ideally, anchoring phenomena for storylines are selected from a list of candidates based on empirical measures of student interest (Penuel et al., 2022a,b). Given constraints inherent to the current design project, which is centered on establishing conceptual linkages to a *prior* learning experience with a primary focus on developmental neuroscience, we did not collect and analyze data on student interest in this anchoring phenomenon *before* its initial selection. However, information on this important aspect of the storyline was collected during mock anchoring phenomenon routines (see Summary and Key Recommendations section) and is currently being collected from early adopters (15 teachers in total) through surveys that are completed after storyline enactment in the high school science classroom.

#### 2. Establishing a storyline framework anchored to mirror movement disorder.

We employed several overlapping strategies to design a preliminary storyline framework anchored to CMM. We reasoned that these processes would act synergistically to aid the design of storyline lessons that not only support scientific discovery and incremental model building, but also student epistemic agency (by addressing a cascading series of questions formulated by students as they incrementally construct explanatory models for the disorder).

##### A. Unpacking the prevailing model for congenital mirror movement disorder.

**Figure 1.**
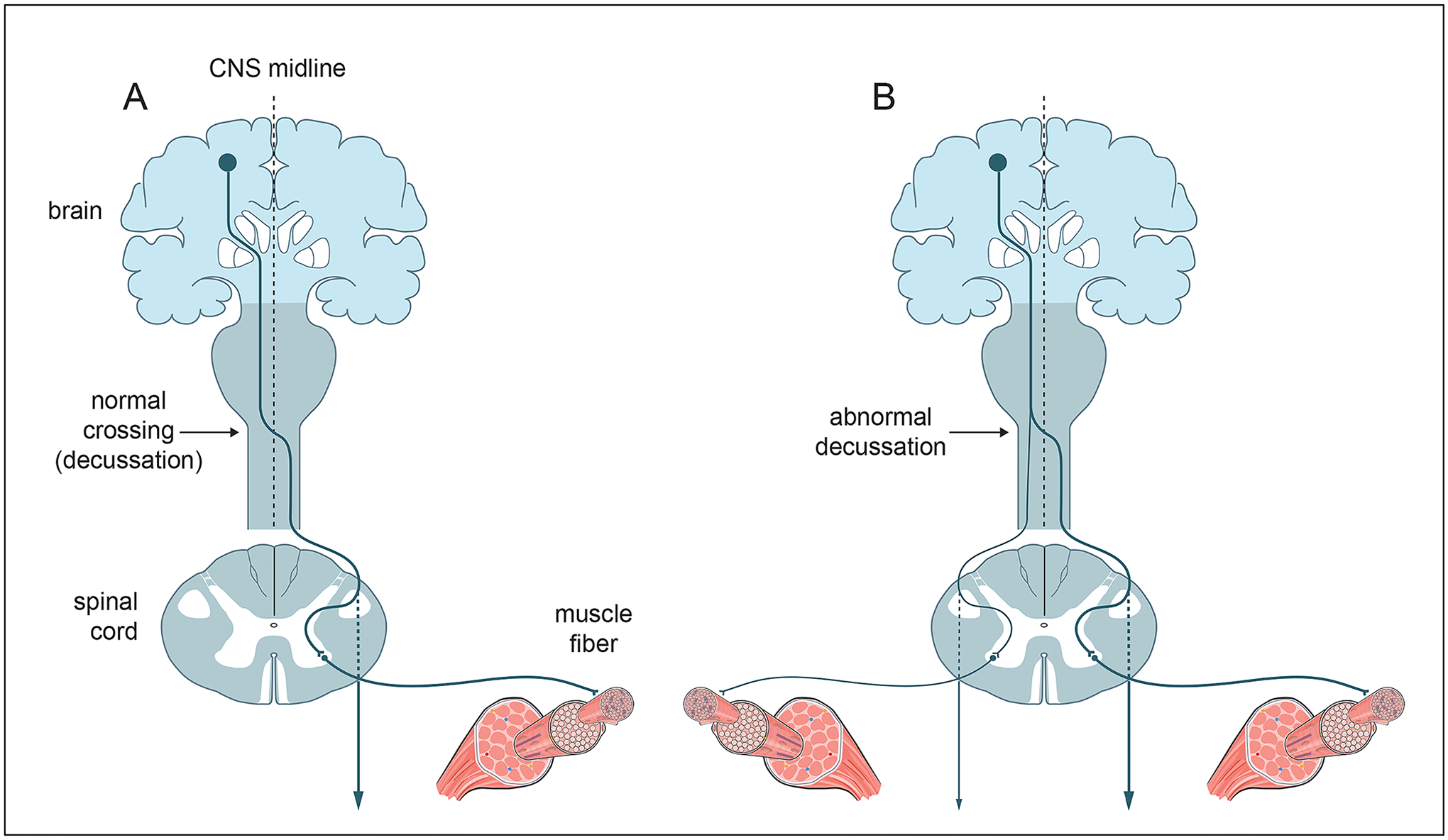
A model showing the corticospinal tract in normal human subjects and patients affected by congenital mirror movement disorder (CMM). In normal human subjects (A), axons from cortical motor neurons in the brain cross the midline of the central nervous system (CNS) at the junction of the medulla and the spinal cord (indicated by arrow) and make connections with α spinal cord neurons on the opposite (contralateral) side of the body axis. These spinal cord neurons activate muscle fibers controlling movement of the hands. In patients affected by CMM (B), some cortical motor axons fail to cross the CNS midline. As a result, these axons establish connections with α spinal cord neurons that activate muscles on the same (ipsilateral) side of the CNS. This neuroanatomical defect, which arises during CNS development due to an axon guidance error involving mutations in either the *NTN1* or *DCC* genes, can be inferred from electrophysiological tests (e.g., electromyelography and transcranial magnetic stimulation) or visualized directly with diffusion tensor imaging tractography.

The currently accepted model for CMM assimilates discoveries made from converging lines of behavioral, neurophysiological, neuroanatomical, genomic, molecular genetic, cellular, and developmental data generated over the last several decades. To familiarize co-designers with the nature of this evidence and help them conceptualize the prevailing model for CMM, the Project Leaders prepared the following technical summary and presented it for group discussion:

Congenital mirror movement disorder (CMM) is characterized by unintentional or involuntary movements on one side of the body axis that mirror, to varying degrees, voluntary movements performed on the contralateral side (with the homologous distal extremity; Cox et al., 2012). Primarily affecting the hands, CMM is a familial disorder with an autosomal dominant mode of inheritance (with incomplete penetrance; Gallea et al., 2011). It is now linked to at least two genes with well-established roles in axon pathfinding: Netrin-1 (*NTN1*), which encodes a secreted axon guidance signaling cue (Pourchet et al., 2021; Méneret et al., 2017; Yamauchi et al., 2017), and Deleted in Colorectal Cancer (*DCC*), which encodes a cell surface receptor for the Netrin-1 signaling protein (Srour et al., 2010; Welniarz et al., 2017; Finger et al., 2002; Jain et al., 2014; Méneret et al., 2014). CMM is also linked to at least one gene involved in regulating intracellular signaling by Netrin-1 (*RAD51*; Glendining et al., 2017; Gallea et al., 2013; Franz et al., 2015; Méneret et al., 2014). Consistent with these findings, the pathophysiology of CMM appears to be related to developmental defects in the formation of the corticospinal tract (which relays signals from the motor cortex to spinal cord motor units/muscle targets on the contralateral side of the body; Fig. 1), and defects in the formation of the corpus callosum (which mediates communication between the motor cortices; Varadarajan et al., 2017; Moreno-Bravo et al., 2019; Marsh et al., 2018).

Based on these and related findings, the group delineated seven core discoveries required for students to incrementally build a comprehensive model of the anchoring phenomenon (Table 3, column 1).

##### B. Eliciting questions about the anchoring phenomenon from students during the co-design process.

As noted elsewhere, lessons comprising a storyline seek to support incremental knowledge building and discovery through investigations that are motivated by student questions about an anchoring phenomenon (Penuel et al., 2022b). Accordingly, a top-tier goal for storyline designers is to anticipate the range of student questions that emerge during a lesson sequence and then modify the lesson sequence after examining actual questions posed by students during pilot storyline enactments (Penuel et al., 2022a,b).

Given the emphasis of storylines on supporting student epistemic agency, we elicited *initial* student questions about the anchoring phenomenon early in the co-design process, *before* the initial lesson flow was delineated and implementation trials of the storyline unit were conducted in the classroom. For this important component of our storyline design process, teacher co-designers presented 79 11th and 12th grade students (with no prior exposure to the program or the anchoring phenomenon) with three videos depicting CMM patients of different ages and ethnicities performing a motor task under the direction of a clinician. Students were then invited to record their questions in a shared online spreadsheet. Because student contributors were enrolled in a range of standard, honors, and career technology education courses, we expected that their questions would provide a reasonably accurate representation of the initial questions posed by students who are subsequently engaged in pilot enactments of the fully developed NeuroLab 2.0 storyline. After questions were de-identified by teachers, the project leaders subsequently conducted a latent thematic analysis of all 349 questions collected from students to expose their early thinking about CMM, identify emergent patterns and themes defined by their questions, and explore possible trajectories of storyline exploration (Table 4).

To perform the analysis, we roughly followed the procedure previously described by Braun and Clarke (2006). Briefly, we began by aggregating the full complement of de-identified student questions into a single row of an Excel worksheet and then deconstructing compound questions into individual component questions (data items), wherever necessary. We then reviewed the resulting list of deconstructed questions several times to better familiarize ourselves with their content/meaning and to compile a running list of key terms that appeared repeatedly within the students’ questions (an iterative and highly time-consuming process). For instance, the terms *neurological*, *brain*, and *nervous system* were repeatedly recognized during our reading of data items along with a number of other terms that we later used to search for, find, and group similar questions (which were assigned an interim identifier or text tag).

In the next phase of our analysis, we examined both categorized and as yet uncategorized questions to identify an additional set of key terms that would aid their assignment to new or more narrowly focused bins. For instance, questions that made explicit reference to specific brain regions (e.g., *How does it affect the frontal lobe and decision making skills?*), brain structures (e.g., *Since the cerebellum is responsible for movement, is something impaired in that area?*), or neural subsystems (e.g., *Does this condition rely on visual perception?*) were sub-grouped under a broader theme that encompassed questions that made general and more specific references to the brain and nervous system. Consistent with the latent nature of this analysis, it often became necessary to interpret the underlying ideas, assumptions, and conceptualizations inherent to certain questions in order to ascertain student meaning and guide their categorization (another extremely time-consuming aspect of the analysis). For instance, a search using the key terms *sensory*, *sensation*, or *visual* would fail to identify the following student question: *If blindfolded, would it make any difference?* However, because this particular student appeared to wonder if the movement behaviors displayed by affected patients were somehow affected by visual input, we grouped this question with other questions that made implicit or explicit references to sensory systems (Table 4; Peripheral, Sympathetic, and Sensory Systems subtheme). After examining questions for content that warranted their assignment to two bins, we exhaustively reviewed the contents of each major category and subcategory until we were confident that we identified and appropriately grouped the major conceptual themes and subthemes represented by student questions. As a final step, we scored the number of questions that were assigned to each thematic category or subcategory.

Interestingly, many of the themes/sub-themes that emerged from students’ initial questions align with basic and clinical research avenues pursued by scientists and clinicians to create the currently accepted model for CMM. Not surprisingly, motor behavior formed the most dominant theme in our analysis of student questions (~ 43% of all questions formulated by students). Over 50% of the questions comprising this theme made explicit reference to the mirroring of hand or limb movements (bimanual synkinesia). Furthermore, ~ 18% of questions that referenced mirror movements also made an unambiguous reference to the voluntary nature of the mirroring, whereas 5% alluded to the intensity of the mirroring. Taken together, these observations indicated widespread student recognition of synkinetic (mirror) movement, the primary clinical feature of CMM and the selected target of incremental model building.

Questions with specific etiological references to the nervous system or its component subsystems and structures accounted for ~ 27% of the total number of questions posed by students. By contrast, a surprisingly small number of questions linked the motor behavior to abnormalities in other body systems—most notably the muscular and skeletal system. The over-representation of questions referencing the nervous system reflected students’ attempts to link defects in nervous system function to the motor behavior observed in the videos (an encouraging finding given the neuroanatomical basis of CMM). We predicted that the number/frequency of questions in less dominant themes would increase as students progress through the storyline pathway during ongoing implementation trials. We also anticipated the emergence of questions that define new themes. Feedback obtained from early adopters is consistent with both predictions (see Section 3 below).

##### C. Mapping initial student questions to core storyline discoveries.

In an effort to examine student questions in the context of core storyline discoveries, we mapped representative questions to each of the seven storyline discoveries that were deconstructed from the prevailing model (Table 3, column 1). Binding storyline discoveries to student questions, irrespective of their overall level of representation/frequency, revealed a high degree of concordance between the themes/subthemes represented by students’ initial questions and the core storyline discoveries essential for incremental model building. We repeated this mapping process again during the construction of component lessons to ensure that the majority of initial questions posed by students were addressed at some point in the storyline sequence (either directly/explicitly or indirectly/implicitly). Importantly, the availability of these questions prevented our group from overlooking questions that students may deem important but that we did not regard as essential for incremental model building. For instance, initial questions about lifestyle impacts or symptom management (Table 4) do not figure prominently in the prevailing model of CMM. However, opportunities for students to explore and answer these questions were provided by semi-fictitious case studies that we compiled during the lesson design process to address other questions critical for student progression through the storyline (see Section F for additional information). The extent to which students recognize that their questions are addressed by the unit (an important indicator of the unit’s coherence) will be examined after the completion of implementation trials that are currently underway with early adopters.

##### D. Selecting evidence and resources to support student discovery.

An important step in the creation of storyline lessons involved the selection of evidence to support student discovery and address student questions. Given their scientific background and expertise, understanding of the current model for CMM, and familiarity with widely utilized scientific tools and resources, this facet of the initial co-design cycle was largely conducted by the project leaders and involved periodic consultation with teacher co-designers. The types and sources of data/evidence selected for inclusion in the storyline trajectory are summarized in Table 3 (columns 2 and 3). These data were selected based on their ability to support the construction of an explanatory model consisting of multiple dimensions. The design of a storyline unit that culminates in the creation of a multidimensional model was intended to help students recognize the interdisciplinary nature of biomedical science, an important feature of educational experiences developed by our organization.

In keeping with our goal to support data literacy and maintain the authenticity of the NeuroLab 1.0 ISE, we intentionally avoided the use of popular media to support student movement through the CMM storyline (e.g., v-logs, blogs/blogposts, podcasts, etc.). Instead, we drew upon scientifically credible sources that are frequently utilized by scientists in their own daily practice to obtain and evaluate data. These sources took one of three primary forms: 1) data figures obtained from published journal articles; 2) clinical case studies; and 3) data records from authoritative databases.

In consultation with a professional illustrator, we adapted data figures selected from peer-reviewed journal publications to reduce their overall complexity and to emphasize (or sometimes exaggerate) elements/components that support a given conclusion or research finding. Wherever possible, the style, colorization, and other artistic features of adapted data figures were kept consistent with illustrations used elsewhere in the storyline to introduce related data or various forms of foundational information (see Section E below). As noted below, for each figure or series of data figures, we embedded visual and text prompts to help guide student analysis and interpretation, and foster recognition of key findings. Prompts were also used to help students relate the findings conveyed by one data figure to evidence that they encountered in other segments of the storyline pathway (see Appendix A). Teachers’ input was especially valuable in creating prompts that not only support student data analysis and interpretation, but also support the execution of specific instructional routines that reflect the storyline approach to student engagement (Reiser et al., 2017).

In addition to data figures adapted from primary research articles, we created multiple opportunities for students to obtain and evauate evidence relevant to the storyline from authoritative online sources, including the Online Mendelian Inheritance in Man database (OMIM; Amberger and Hamosh, 2017) and the NCBI-ClinVar platform (Landrum et al., 2014; Landrum et al., 2016; Landrum et al., 2018; Landrum et al., 2020). Managed by professional curators, the data records contained in these platforms aggregate, organize, and summarize evidence mined from peer-reviewed scientific journal articles. They also provide links to interoperable databases and other online resources that contain related forms of information pertinent to students’ model-building mission. To support student navigation through these data repositories, we created multimedia instructional resources for whole-class use that make extensive use of text prompts, web page screenshots, and expanded views of various page features and elements (see Appendix B). Additional prompts were embedded into these resources to narrow students’ focus on evidence and information most relevant to storyline discovery and incremental model building.

During this phase of the project, we also selected interactive informatics tools to support student exploration. Through the guided use of these tools, students are able to: 1) diagnose the movement disorder using clinical features and other forms of information presented in semi-fictitious case studies (Human Phenotype Ontology Database; Köhler et al., 2021); 2) visualize spatially organized neuroanatomical and gene expression data (Allen Brain Map/Brain Explorer 2.0 [Lau et al., 2008], OpenWorm [Szigeti et al., 2014]), examine and manipulate gene variant sequence data (EMBOSS Transeq [Rice et al., 2000], Clustal Omega [Sievers et al., 2011; Sievers and Higgins, 2014]); and 3) explore physical interactions between axon guidance proteins implicated in the movement disorder (Simple Modular Architecture Research Tool [SMART; Letunic and Bork, 2018; Letunic et al., 2021]). The engagement of students in the use of these resources constitute *in silico* exploratory labs that we distinguish from more familiar wet labs associated with life science experiences. Apart from their role in facilitating student discovery and incremental model building, in silico labs are intended to support (big) data literacy and highlight data sharing as an important dimension of scientific collaboration in the biomedical sciences.

##### E. Identification of requisite foundational knowledge to support data analysis and interpretation.

Students’ ability to competently analyze and interpret data that supports storyline discovery and incremental model building is reliant on their understanding of various types of foundational information. Teachers began taking stock of this basic information after the core storyline discoveries essential for incremental model building were articulated (Table 3, column 1). For instance, the analysis and interpretation of neurophysiological data that we selected for inclusion in the storyline (e.g., electromyelography and transcranial magnetic stimulation) require a basic understanding of how activity is transmitted through neural pathways via the synapse or neuromuscular junction. In a similar vein, the analysis and interpretation of gene variants linked to CMM require a basic understanding of the gene concept and the processes of transcription and translation. As noted in Section G below, the identification of foundational information also revealed a number of opportunities to address NGSS elements rooted in life science topics.

##### F. Lesson creation (key design considerations).

Lesson design conformed to Universal Design for Learning Guidelines (CAST, 2018), wherever possible. The overarching goal of this project phase was to create a suite of classroom resources that elicit a cascading series of student questions and a progression of corresponding themes that align with the core storyline discoveries (Table 3, column 1). In this section, we outline a number of specific strategies that we employed during the lesson creation phase of the project to effectively guide students through the NeuroLab 2.0 storyline while maintaining the continuity and coherence of the learning experience. A brief synopsis of each lesson is presented in Table 5.

### *Just enough* approach to the introduction of content.

During our co-design process, the selection of foundational content exposed some sharp divisions of opinion regarding the depth and breadth of information that students should encounter during the storyline. To support coherence from the students’ viewpoint, we avoided the introduction of storyline content that exceeds what is necessary for incremental model building (a sometimes difficult judgment call that required group discussion). Although the introduction of foundational information is undoubtedly necessary for orienting students to a relevant model organism (e.g., human, mouse, or nematode worm), model system (e.g., the developing spinal cord or ventral nerve cord) and the scientific findings obtained through its study (e.g., axon projection errors), attention was paid to the level of detail and depth with which this information is encountered by students. For instance, the motor behavior that drives the CMM storyline (bimanual synkinesia) is caused by a neurodevelopmental defect in an important but relatively straightforward neural circuit that connects motor neurons in the brain to contralateral spinal cord neurons that innervate/activate muscle fibers (Fig. 1). Although a comprehensive survey of the nervous system and its various subdivisions may be necessary or appropriate for meeting the learning objectives of an undergraduate neuroscience course or a high school Advanced Placement Psychology course, it is unlikely to benefit students’ advancement through the NeuroLab 2.0 storyline and directly support our goal of fostering student proficiency in the application of science practices toward model building. To the contrary, the extraneous detail is likely to divert focus to ideas and concepts that are uncoupled from core storyline discoveries and the preponderance of questions formulated by students.

To accommodate our *just enough* approach to storyline content, it became necessary to not only develop classroom materials that withhold/omit superfluous information and detail that does not significantly advance the storyline, but also to control the *timing* of content introduction in the lesson sequence so that it roughly follows (anticipates) the emergence of student questions. These design considerations impose a number of practical challenges associated with the use of existing open-access resources available to teachers and students through various sources. To overcome these challenges, we invested significant time in the creation of customized resources and graphics that were specifically designed to focus students on ideas, concepts, and data most relevant to their questions and emerging conceptual models. In a similar vein, the pathways taken by students to navigate within authoritative databases (see Appendix B) required careful planning so as to avoid, inasmuch as possible, unstructured forays into virtual spaces where superfluous or precocious data encounters were inevitable. In the current storyline, navigation in OMIM, ClinVar, and other platforms was carefully structured so that student movement terminates at standard format data records containing information that students are guided to evaluate and connect with related information that they encountered elsewhere in the NeuroLab 2.0 storyline. It is important to note here that the integration of database navigation into the storyline experience will require our team to make periodic revisions to classroom materials that reflect changes made to various platforms during routine updates.

### *Just-in-time* approach to the introduction of content.

We also encountered significant tension when discouraging the common practice of *front-loading* content (especially foundational content that aligns with life science course objectives). From our perspective, exposing students to ideas and concepts outside of the storyline implementation window disrupts coherence by uncoupling exploration from student questioning and undermining the *need to know* that arises as students navigate through the storyline and identify gaps in their emerging explanations. To safeguard coherence, lesson design was guided, in part, by a *just-in-time* philosophy that provided an opportunity for students to explore related life science ideas and concepts within the framework of the storyline itself. As noted by co-designers, this facet of unit design will require teachers to restructure their semester or course plans to fill the gaps created by the movement of a particular course topic into the storyline implementation window.

### Using models to support incremental model building.

Materials created for whole-class use made pervasive use of graphical models to support students in the practice of *using* models while conveying important ideas and concepts relevant to *building* models. Inasmuch as possible, graphics were specifically designed to frame foundational ideas and concepts within the very same systems that form the focus of storyline explorations. For instance, models depicting the action potential and the propagation of neural activity were introduced in Lesson 2 to help students analyze and interpret electrophysiology data that they encounter in Lesson 3 (Table 5). Rather than utilizing generic representations of interacting neurons found in many science textbooks to introduce this important process, our models utilized neurons comprising the motor pathway that is directly affected in CMM patients (e.g., the innervation of lower spinal cord neurons by upper cortical motor neurons via the synapse, and the innervation of muscle cells/fibers by lower spinal cord neurons via the neuromuscular junction). In a similar vein, the processes of cell division (neurogenesis) and differentiation (neuronal cell fate specification) were rooted in the systems implicated in the movement disorder (e.g., the developing neural tube).

### Embedding prompts into instructional materials.

Each lesson is accompanied by a multimedia presentation that is intended to engage the entire class in interactive discussions centered on storyline exploration and model building. For these resources, we made extensive use of embedded prompts to support teachers in the execution of storyline instructional routines, ask students questions pertinent to an emerging idea or concept, facilitate the interpretation of graphical models, engage students in the analysis and interpretation of storyline data, stimulate discussions on linkages that exist between different concepts or between different types of data or findings, and encourage dialog about emerging models (see Appendix A). As noted above, visual and text prompts were also used to help students navigate within authoritative databases to find data and information relevant to their explanations (see Appendix B).

### G. Identify/unpack NGSS elements encompassed by the storyline (retrospectively and en route to lesson co-creation).

Given our goal to adapt a pre-existing learning experience for high school course integration, the group established student engagement in investigational practices (i.e., NGSS science practices) as an immediate project priority or *pro tem* goal, irrespective of the specific performance expectations, disciplinary core ideas, and crosscutting concepts encompassed by the emerging unit. Accordingly, the bundling and unpacking of related standards that often occurs during the initial design of storyline units was intentionally deferred. Our expectation was that movement through the co-design process toward the creation of lessons that support storyline exploration and discovery would expose related NGSS elements encompassed by the emerging unit, particularly those that address core topics targeted by the NGSS, including *HS.Structure and Function* and *HS.Inheritance and Variation of Traits*. We also anticipated that during lesson creation, we would identify opportunities to address additional NGSS elements without introducing major deviations from the storyline trajectory that jeopardize its continuity or coherence. For instance, students explore the role of two genes (*NTN1* and *DCC*) in an important phase of nervous system development in which the neuroanatomical defect in CMM patients is presumed to arise. To help orient students to the model organisms, model systems, and data that they will interpret and ultimately generalize to CMM patients, we initially created foundational resources for students to explore axon pathfinding, a critical neurodevelopmental process that is partially disrupted in individuals affected by CMM. During the creation of these resources, we recognized an opportunity for students to explore two phases of nervous system development that immediately precede (or overlap) with the process of axon pathfinding, namely neurogenesis and cell fate specification. The inclusion of foundational content related to these neurodevelopmental processes created an opportunity to address, at least in part, HS-LS1–1 (use a model to illustrate the role of cellular division [mitosis] and differentiation in producing and maintaining complex organisms). The complete set of NGSS performance expectations addressed (at least in part) by the NeuroLab 2.0 unit are presented in Table 6. The identification of these NGSS elements created a starting point for the group to articulate lesson and unit-level assessment opportunities later in the design process (see Appendices C and D).

#### 3. Soliciting early teacher feedback on the lesson sequence and its ability to motivate student questions that drive incremental model building.

As noted above, *initial* student questions about the anchoring phenomenon were elicited early in the design process during a mock anchoring phenomenon routine carried-out by teacher co-designers. The availability of this information enabled our team to develop a storyline unit that directly or indirectly addresses student questions (and thereby supports coherency from the students’ perspective). However, we were particularly interested in knowing the extent to which the lesson sequence addressed questions that emerged *later* in the storyline, after students have been introduced to the anchoring phenomenon and have begun to pursue their investigations. We were also interested in knowing if movement through the storyline resulted in the creation of interim models that assimilate data and discoveries that students encountered across lessons.

Survey information obtained from teachers who are conducting ongoing implementation trials support the notion that the lesson sequence is capable of generating a succession of additional questions that lead to—and are addressed by—storyline investigations. As one teacher noted, *[t]he questions moved from general considerations of the body systems involved to more precise questions about how neurons work and how the nervous system develops during embryogenesis, including gene regulation and cell signaling*. Teachers also documented specific examples of emergent student questions that are consistent with this claim. Importantly, these questions were either underrepresented in, or completely absent from, our library of initial student questions (see Table 4 for representative *initial* questions):

- At what embryonic stage do neurons and the corticospinal tract develop?
- During development does all decussation occur at the same time? Or slowly over time?
- Could the cause of the movement disorder be maybe a protein that was not made or not made properly?
- What proteins affect decussation?
- At what stage of development do proteins that are coded for decussation become expressed?
- What does Netrin-1 protein bind to?
- Does the mutation create a different amino acid, or does it create a stop codon?
- Is it the ligand that is misshapen or the receptor?

**Figure 2.**
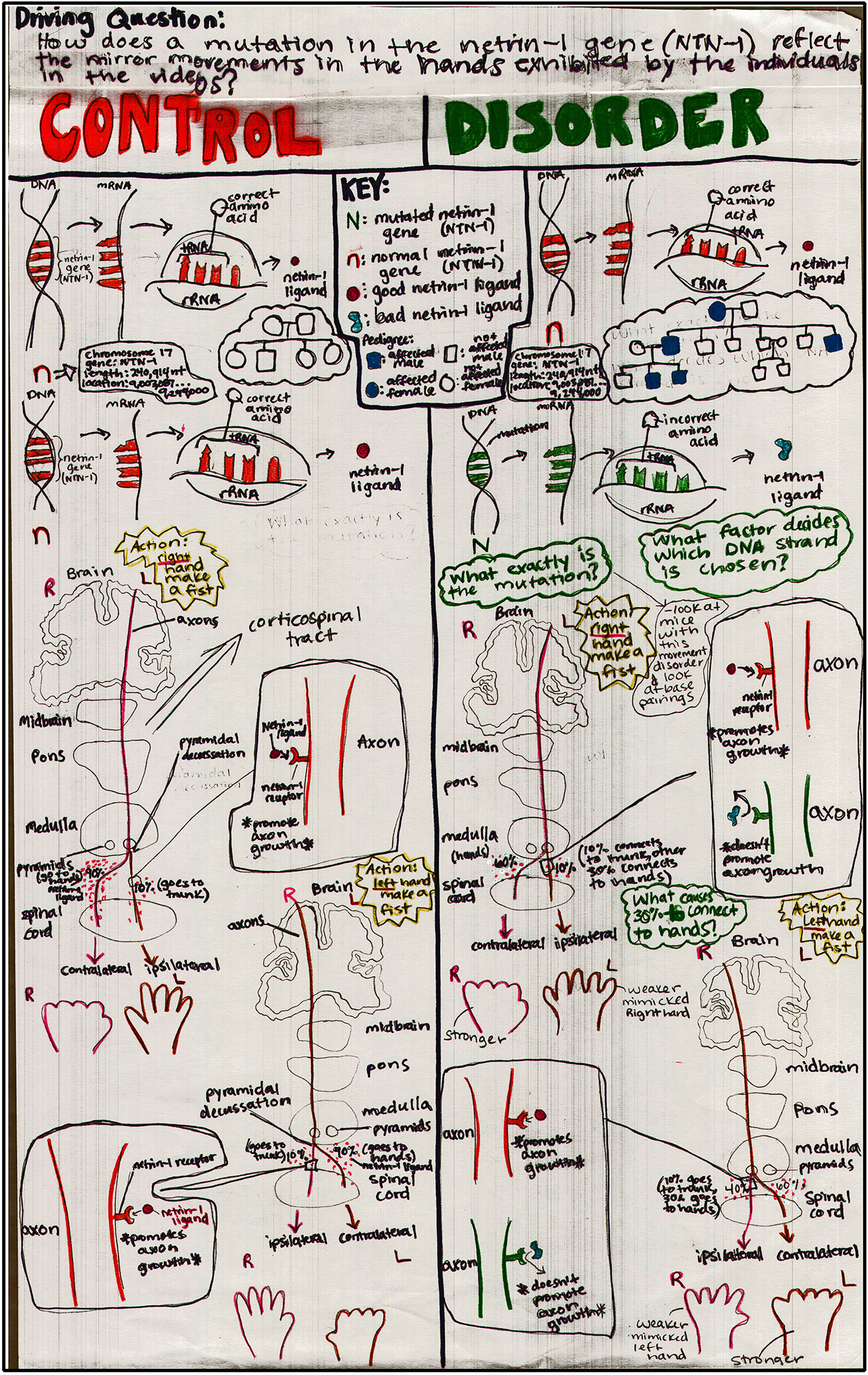
Interim model created by a student during an early implementation trial of the storyline.

A brief examination of de-identified models created by students during these early trials (see Fig. 2 for an example) also supports the ability of the lesson sequence—and the evidence it introduces—to promote continued movement through the storyline pathway (e.g., students assimilated target discoveries into their models and appeared to make appropriate model revisions based on their recognition of gaps in preceding models and their exposure to new data/evidence). A structured examination of successive models created by students during implementation trials will form an important component of our ongoing efficacy study. We will also examine the progression of question themes that emerge over time as students advance through the storyline pathway and make iterations to the lesson flow wherever warranted.

## SUMMARY AND KEY RECOMMENDATIONS

The collaborative approach that we adopted for storyline design is rooted in the same theoretical frameworks and guided by the same principles as published strategies for the *de novo* design of storyline units (Penuel et al., 2022a,b; Nordine et al., 2019). However, our approach creates a potentially attractive design alternative for scientists seeking to bring aspects of their own research—or to adapt elements of existing education projects under their leadership—into high school science classrooms (see Table 2). We conclude this case study by highlighting several broad recommendations that emerged from our co-design work.

### Prioritize the selection of biomedical phenomena over the identification of NGSS performance expectations at project inception.

Biomedical researchers seeking to co-create STEM+M storyline units that address NGSS performance expectations may find it useful to defer the identification/discussion of NGSS performance expectations and instead begin the design process by considering potentially engaging phenomena that align with the primary focus of their basic, clinical, or epidemiological research. It seems reasonable to make two key assumptions about biomedical phenomena with respect to storyline design: 1) they create a rather expansive list of storyline anchors of potential interest to students given their emphasis on human health issues that are likely to affect someone in their family or community; and 2) prevailing models of these phenomena have an extremely high probability of aligning with core topics targeted by the NGSS, including *HS.Structure and Function* and *HS.Inheritance and Variation of Traits*. Furthermore, deconstructing models of biomedical phenomenon into their component discoveries early in the design process (Table 3, column 1) will enable design teams to expose and discuss related NGSS ideas and concepts and the degree to which a given performance expectation can be reasonably addressed by a storyline organized around the selected anchor (Table 6). In our experience, the creation of lessons will present exciting opportunities for the design team to consider the development of potentially new and unanticipated storyline segments that support features of specific NGSS performance expectations (see Appendix C).

### Solicit early feedback on student interest in the selected anchoring phenomenon.

For more traditional approaches to storyline design, the selection of an anchoring phenomenon is more open-ended and driven by a number of considerations that, in some cases, include empirical measures of student interest (Penuel et al., 2022a). Our choices for an anchoring phenomenon (CMM) were severely constrained by our project goal to link a major conceptual focus of the NeuroLab 1.0 ISE (midline axon pathfinding) to the new storyline. Because scientists seeking to adapt a pre-existing experience will undoubtedly face similar limitations, their initial selection of a storyline anchor is likely to occur in the absence of information on student interest in the chosen phenomenon. Before proceeding to the next step of the storyline design sequence, we strongly recommend that designers solicit some form of feedback on student interest immediately after selecting an anchoring phenomenon—or candidate anchoring phenomena (via brief student interest surveys and/or indirect feedback from teachers). The mock anchoring phenomenon routines conducted by our teacher co-designers enabled our group to obtain preliminary feedback about students’ level of interest in the disorder (via short teacher surveys). Though indirect, the positive feedback we obtained from these surveys provided the justification for proceeding through a rather lengthy co-design sequence with our teacher partners.

### Engage students in the co-design process.

As noted above, in addition to *anticipating* the range of questions that students might ask about the movement disorder, teachers on our team conducted mock anchoring phenomenon routines in their classrooms during the initial design process to elicit actual students’ questions about the movement disorder. From the standpoint of student coherence (Penuel et al., 2022b), this is an indispensable storyline design element that development teams can readily employ to directly involve students in the co-design process. The questions posed by students can be organized into themes/subthemes using the method described in this report and then mapped onto core storyline discoveries to ensure that the emerging lesson sequence responds to initial student questions about the movement disorder.

In addition to collecting initial student questions during the execution of mock anchoring phenomenon routines, we recommend that teacher co-designers (and early adopters) create a record of student questions that emerge during early storyline implementation trials conducted in the classroom. A detailed examination of these data (across lessons and classes) will enable the development team to evaluate the extent to which the storyline is responsive to more specific and narrowly focused questions that students pose later in storyline pathway. Early adopters who are conducting implementation trials of the NeuroLab 2.0 storyline are currently monitoring the emergence/timing of student questions in relation to the lesson sequence and the specific forms of evidence that students encounter. We plan to conduct detailed analyses of these questions to identify themes that emerge as students progress through the storyline and their congruency with the themes defined by core storyline discoveries that students make to construct their models. These analyses are likely to inform changes to the storyline lesson sequence that enhance its coherence. Changes will be made in an iterative fashion during learning community meetings that follow each implementation cycle.

Given the emphasis of storyline units on supporting epistemic agency and coherence from the students’ perspective, it seems reasonable to propose that students can play an even bigger role in the storyline design process, *before* implementation trials commence in the science classroom. We are currently exploring a framework whereby students are not only introduced to the anchoring phenomenon early in the design process, but also to the key discoveries unpacked from the prevailing conceptual model of a phenomenon. According to this approach, questions would be elicited from students during a one- to two-week design trial conducted by teacher co-designers. During the pilot trial, teachers would not only record questions formulated by students after observing the anchoring phenomenon, but questions that arise after students are introduced to each of the major storyline discoveries essential for incremental model building. These discoveries can be presented sequentially in the form of customized infographics that combine text descriptions of each storyline discovery with supporting images and illustrations.

### Provide opportunities for peer-centered discussions around teachers’ concerns and uncertainties.

The NeuroLab 2.0 storyline makes extensive use of databases to engage students in science practices and support discovery-making critical for incremental model building. Although teachers uniformly recognized database navigation as an important mechanism for students to find and analyze data relevant to their evolving model, they expressed some initial concerns over *losing the class* during in silico labs. This general concern—which may act more broadly to discourage uptake of programs seeking to promote student data literacy in the classroom—appeared to be rooted in two chief issues: 1) managing the movement of an entire class of students through various databases (a concern that factored prominently in the design of some lesson resources) and 2) a perceived inability of students to recognize these data-centered experiences as being integral to the daily practice of biomedical science and complementary to the wet lab experiences that are typically associated with the work of a scientist.

Given our just-in time philosophy with respect to the introduction of science content, some teachers also expressed concerns over the time needed for adopters to restructure course plans in order to move their normal coverage of course content into the storyline implementation window. Uncertainty over their students’ reactions to a new way of experiencing science and an unfamiliar way of interacting with classmates and being assessed was also verbalized by some group members. Although our co-design phase was grounded in the joint creation of storyline unit resources, we allocated ample time for teachers to engage in peer-focused discussions centered around potential strategies and interventions to address these concerns.

### Develop storyline-specific resources to support teachers in guiding their students through the unit.

Implementation of the NeuroLab 2.0 storyline unit requires a radically different approach to science instruction than *business-as-usual* teaching practices. Accordingly, our intentional design of a storyline that conforms to well-conceived principles rooted in the learning sciences does not ensure that it will achieve its intended student learning outcomes. Although a number of generic instructional strategies/routines have been articulated by storyline developers to help teachers effectively guide their students through storyline units (Penuel et al., 2022a,b; Reiser et al., 2017), we suggest the creation of complementary and project-specific teacher resources that are directly tied to the questions, evidence, and key discoveries that students pursue or use to create their explanatory models. Table 3 provides an adapted version of a teacher resource that we developed to support teachers in the execution of several general instructional routines advocated for storyline enactment. Additional materials will be discussed elsewhere in the context of our professional development programming along with efficacy data that we are currently collecting from early adopters and their students. All materials and resources developed in connection with the NeuroLab 2.0 project will be made openly available to the broader educational community upon project completion.

## Supplementary Material

S1

S2

S3

S4

## Figures and Tables

**Table 1.** Unit co-design timeline and activities

Design Segment	Duration/Dates	Primary Group Activities
**Initial Summer Institute**	**5 consecutive days** 07/29/2019 to 08/02/2019	Establish the objectives and goals of NeuroLab project and their alignment with the goals of the NIH SEPA program Clarify the roles and responsibilities of Project Leaders (PIs), Project Coordinator, and Teacher Co-Designers Deepen teacher understanding of the ideas, concepts, and methods encompassed by the NeuroLab 1.0 ISE experience and its targeted educational outcomes
**Unit Co-Design Workshops**	**12 one-day workshops** 09/13/2019 10/17/2019 12/08/2019 02/02/2020 06/23/2020 07/15/2020 08/30/2020 11/15/2020 08/30/2020 11/15/2020 03/07/2021 05/23/2021	Reach group consensus on a unit design strategy for NeuroLab 2.0 Build group knowledge around the storyline approach to unit design and its ties to the NRC Framework and NGSS Develop ideas for an anchoring phenomenon of potential interest to students and connected to the axon pathfinding phase of neurodevelopment (a major focus of the NeuroLab 1.0 ISE) Unpack key storyline discoveries from technical description of the movement disorder Select data/evidence to support storyline discovery Identify foundational information required for students to analyze and interpret data selected for discovery Generate a list of questions anticipated from students after being introduced to movement disorder Conduct a mock anchoring phenomenon routine in several classrooms to elicit actual questions from students Analyze student questions to identify themes and map to key storyline discoveries Delineate a lesson sequence that supports incremental model building and responds to actual questions elicited from students during mock anchoring phenomenon routines conducted by co-designers in the classroom Identify NGSS elements encompassed by emerging unit
**Post-Workshop Summer Institute**	**5 consecutive days** 07/28/2021 to 08/01/2021	Review assembled unit (by lesson) and make revisions to improve coherence and enhance student understanding of ideas, concepts, and data encountered in the sequence Identify points in the storyline for students to create interim explanatory models Conduct (truncated) implementation trials to elicit actual questions posed by students later in the storyline and to informally evaluate early models for evidence of student understanding of storyline data (conducted in May 2022, after co-design workshops concluded and before the first cohort of new teachers participated in PD experiences hosted in summer 2022)
**Concluding Workshops**	**2 one-day workshops** 01/30/2022 02/06/2022	

**Abbreviations:** ISE, informal science experience; NIH, National Institutes of Health; NGSS, Next Generation Science Standards; NRC, National Research Council; PD, professional development; PI, primary investigator; SEPA, Science Education Partnership Award program

**Table 2.** Storyline design sequence used to translate an original ISE experience into a storyline unit

	Co-Design Element	Primary Contributors
*Identify/unpack NGSS elements en route to lesson co-creation*	*Pre-select a biomedical phenomenon of potential interest to students and tied to major conceptual focus of NeurLab 1.0 ISE*	Entire group
	*Unpack (deconstruct) the prevailing model of the phenomenon into its component scientific discoveries*	Project Leaders
	*Conduct a mock anchoring phenomenon routine with students to elicit initial questions and assess interest*	Teacher Co-Designers
	*Analyze student questions for patterns and themes*	Project Leaders
	*Map themes defined by student questions onto themes defined by core storyline discoveries*	Project Leaders
	*Select data and evidence to support students in making core storyline discoveries*	Project Leaders
	*Identify foundational information that students need to learn or review in order to interpret storyline data and make discoveries*	Teacher Co-Designers
	*Co-create a lesson sequence with associated questions*	Entire group
	*Identify/unpack NGSS elements encompassed by storyline (retrospectively and en route to lesson co-creation)*	Entire group
	*Modify lesson sequence based on the emergence of student questions and progression of corresponding themes during implementation trials (to be carried-out after several implementation cycles by early adopters)*	Entire group

**Table 3.** Storyline discoveries, related student questions, supporting evidence, and sources of supporting evidence organized by lesson.

Discovery that students assimilate into model	Supporting evidence analyzed, interpreted, or generated by students	Source of evidence or information	Lesson (L)
Individuals of different ages and ethnicities are affected by a rare abnormality in voluntary movement (motor **behavior**). For these individuals, voluntary movement in one hand or foot is accompanied by involuntary movement in the opposite (contralateral) hand or foot (bimanual synkinesia). Links to student questions centered on behavior: *Why does he have a hard time controlling his hands?* *Are the hands copying each other?* *Does this child always move both hands in the same way when doing one-handed activities?*	Supported by students’ observations of individuals performing motor tasks in a clinical setting	Videos of individuals affected by the movement disorder (some presented as supplemental data in a peer-reviewed journal publication)	L1
	Supported by descriptions of **behavior** documented by scientists and clinicians	Semi-fictitious case reports of family members affected by the movement disorder (adapted from reports that appear in peer-reviewed journal publications)	L5A2
		Data records from the Online Mendelian Inheritance in Man (OMIM) database and links contained therein	L6A-6C
The abnormality is linked to a **neuroanatomical** defect in the corticospinal tract, a descending pathway that connects motor neurons in the brain (upper motor neurons) to lower motor neurons in the spinal cord, to muscle fibers. Links to student questions centered on body systems and the interaction of the muscular system with the nervous system: *Is this disorder associated with the brain or is it a muscular issue?* *Are the nerves in the hands connected in a way they shouldn’t be?* *Is the opposite side of the brain controlling the other side of the body’s movements?*	Inferred from (suggested by) functional magnetic resonance imaging data and the results of **electrophysiological** tests (electromyelography, transcranial magnetic stimulation) performed on human subjects affected by the disorder	Select data figures and graphs adapted from peer-reviewed journal publications	L3A, 3B
	Supported by phenotypic information displayed in the Online Mendelian Inheritance in Man (OMIM) database	Data that students access through the Phenotypic Series tab of an OMIM data record (e.g., the Neurologic phenotype section of clinical synopses table)	L6A-6C
	Demonstrated by diffusion tensor image (DTI) tractography performed on human subjects affected by the movement disorder	Select data figures and graphs adapted from peer-reviewed journal articles	L9A
The neuroanatomical defect occurs during the **development** of the central nervous system (CNS), when nerve **cells** (neurons) seek out target cells with which to form functional connections (axon pathfinding). Links to student questions centered on development: *Are there issues in neural pathways while they’re being formed?*	Inferred from (suggested by) studies of axon pathfinding performed in vertebrate and invertebrate model organisms	Select data figures adapted from peer-reviewed journal articles	L8A-8C
	Supported by phenotypic information displayed in the OMIM database	Data records that students access through the Phenotypic Series tab of an OMIM record (gene links that appear in clinical synopses table open records that provide text descriptions of gene function)	L6A-6C
During CNS **development**, the axons/growth cones of some upper motor neurons fail to respond to a secreted navigational cue that normally guides their crossing over (decussation) to the other side of the midline. Links to student questions centered on development: *Are there issues in neural pathways while they’re being formed?*	Inferred from (suggested by) studies that show axon crossing defects in nematodes with mutations in the *NTN1* ortholog (*unc6*) or *DCC* ortholog (*unc40*)	Select data figures adapted from peer-reviewed journal articles	L8A, 8B
		Open Worm platform (neuroinformatics software that displays wild-type trajectories of axons affected by mutations)	L8B
	Inferred from (suggested by) studies that show axon crossing defects in the hindbrain of mouse embryos with mutations in the *NTN1* gene	Select data figures adapted from peer-reviewed journal articles	L8C
	Supported by the spatially organized expression of Netrin-1 (unc-6) and DCC (unc40) proteins in nematodes and mouse embryos	Select data figures adapted from peer-reviewed journal articles	L8A-8C
	Supported by expression of *NTN1* and *DCC* transcripts	mRNA expression analysis performed by students using data contained in the Allen Developing Mouse Brain Atlas (visualized in 3D using the Brain Explorer 2.0 companion app)	L10A
For affected individuals, the failure of cortical motor axons to respond to the navigational cue and cross the midline is linked to a heritable **genetic** mutation in the either the *NTN1* or *DCC gene* Links to student questions centered on **genetics** and heritability: *Is this a rare birth trait that some people inherit from their ancestors?*	Supported by a pedigree analysis that students perform in the classroom to determine the disorder’s mode/pattern of inheritance	Semi-fictitious case reports of family members affected by the movement disorder (adapted from select reports that appear in peer-reviewed journal publications)	L5A2, 5B
	Supported by a differential diagnosis that students perform in the classroom using the disorder’s clinical features and mode of inheritance	Semi-fictitious case reports of family members affected by the movement disorder (adapted from select reports that appear in peer-reviewed journal publications)	L5A2, 5B
		Diagnosis report generated by the Phenomizer app of the Human Phenotype Ontology platform and links to OMIM data records that appear in sections of the diagnosis report (Phenotypic Series table of OMIM records)	L5C, 6A-6C
Mutations in the *NTN1 gene* may result in the production of a signal protein that is either misfolded and rendered non-functional, or unable to interact with the extracellular matrix due to an amino acid substitution in its C-terminus (**molecular, sub-molecular and inter-molecular** disruptions). Mutations in the *DCC* gene produce truncations in the corresponding signal receptor protein that prevent it from binding to the *NTN1* gene product or transducing a signal inside the growth cone after ligand binding (**molecular, sub-molecular, and inter-molecular disruptions**). Links to student questions centered on genetics *Is this caused by a genetic mutation?* *Is there a particular gene this is associated with?* *How do patients develop this disease? Is it genetic?*	Deduced by students after examining the nucleotide and amino acid sequences of *NTN1* variants using informatics tools (e.g., EMBOSS Omega and EMBOSS Transeq) and relating their findings to the domain structure of the Netrin-1 protein	Data reports in the ClinVar archive that display Pathogenic *NTN1* variants	Lesson 9B
		Data records and protein domain features displayed in the Simple Modular Architecture Research Tool (SMART)	Lesson 9B
	Deduced by students after examining the nucleotide and amino acid sequences of *DCC* variants using informatics tools (e.g., EMBOSS Omega and EMBOSS Transeq) and relating their findings to the domain structure of the DCC protein	Data reports in the ClinVar archive that display Pathogenic *DCC* variants	Lesson 9C
		Data records and protein domain features displayed in SMART	Lesson 9C

**Table 4.** Representative student questions about the movement disorder (anchoring phenomenon) organized by theme/subtheme.

Theme	Count	Example question
**Motor Behavior**		
Mirror Movement	77	*Why does the little boy make the same movements with both hands when it is only necessary to do it with one?*
Volitional Movement	14	*Can he control the motions with focus, or is it completely involuntarily?*
Mirroring Intensity	4	*Why is this person moving one hand much more than the other?*
Abnormal Movement w/o reference to mirroring	39	*Why does he have a hard time controlling his hands?*
Extremities Affected by Mirroring	5	*What other limbs or body parts may also be affected? Feet, toes?*
Motor Task Procedure	10	*Is she being instructed not to move one hand or is she voluntarily moving both?*
**Nervous System**		
Neurological or Nervous System	9	*Does he have a neurological issue that prevents him from doing some tasks with ease?*
Brain and/or Component Structures	46	*Is this disorder due to an abnormality in the cerebellum or damage done to it?*
Spinal Cord	3	*Could this issue be due to a problem in the spinal cord and how directions are sent to parts of the body?*
Motor System	2	*Does she have a motor function issue that prevents her from doing hand motions without causing disruption in her other hand and foot?*
Peripheral, Sympathetic, and Sensory Systems	9	*Is this disorder a problem with the brain, peripheral nervous system, or both?*
Nerves and Neural Pathways	13	*[D]oes this condition have to do with nerves and signals?*
Neural Connectivity	5	*Does this disorder entail an abnormality in which the nerves of one hand is connected or even intertwined with the nerves in the other?*
Neurotransmission	6	*Do the neuron signals which are supposed to only travel to one area, travel to both hands?*
Neurons	5	*Does it have to relate with neuron functions?*
Cognition or Affect	9	*What are the mental processes these people have that made them act a certain way?*
**Muscular System**	5	*Is this a disorder with the muscles in the hand being twitchy?*
**Skeletal System**	1	*[I]s this a malfunction of the nervous system, muscular system, or skeletal?*
**Clinical Issues**		
Diagnosis	18	*Does the person have OCD, ADHD, or ticks?*
Onset	5	*How long has this been noticeable in these patients?*
Progression	8	*[W]ill the “hand stutters” get worse as the child ages?*
Prevalence	3	*[H]ow many people are affected by this?*
Management and Treatment	24	*Could this ever be reversed with physical therapy?*
Etiology	13	*Is this condition the result of trauma or a medical disorder?*
Other Clinical Features	16	*Are there other symptoms like speech, thinking, etc?*
Patient Information	3	*How old is the child?*
**Lifestyle impacts**	8	*How does this affect everyday life?*
**Genetics/Heritability**	13	*Is this a rare birth trait that some people inherit from their ancestors?*
**Development**	3	*Are there issues in neural pathways while they’re being formed?*

**Table 5.** The NeuroLab 2.0 storyline lesson sequence.

Lesson	Focus of Student Exploration	Model Version
**1**	**Observing the behavior of individuals affected by a rare movement disorder (introduction to the anchoring phenomenon)**	**v.1**
During this two-part opening lesson (L1A-L1B), students explore the characteristics and roles of scientific models and observe videos of individuals performing a motor task in a clinical setting (introduction to the anchoring phenomenon). Teachers then invite students to formulate questions based on their observations of the motor behavior and any prior knowledge of disorders affecting human movement. Students subsequently organize their questions into categories that define areas of investigation and exploration. Teachers then invite students to prioritize investigations. At the conclusion of the lesson, teachers invite students to create an initial explanatory model (v.1) that incorporates their observations and prior knowledge/experience. Students are informed that they will have multiple opportunities to revise their model based on the discoveries they make in subsequent lessons. The final model (v.6) will be the target of summative assessment in Lesson (L10).		
**2**	**Exploring the body systems involved in human movement (foundational)**	**v.1**
In L2A, students explore the hierarchical organization of muscle tissue as a first step toward examining the process of muscle fiber activation, which converges on the sliding filament model of muscle contraction. In the second half of this lesson (L2B), students examine the process by which nerve cells within the motor cortex of the brain (upper motor neurons) activate motor neurons in the spinal cord (via the synapse), and the process by which motor neurons in the spinal cord activate muscle fibers/cells (via the neuromuscular junction) to produce contraction.		
**3**	**Interpreting the results of diagnostic tests of movement**	**v.2**
In L3A-L3C, students analyze and interpret clinical data obtained from CMM patients using electromyelography, transcranial magnetic stimulation, and functional magnetic resonance imaging. By connecting this data with foundational information obtained in L2A-L2B and direct observations of the abnormal motor behavior displayed by CMM patients (L1), students discover that the movement disorder is likely to involve a failure of axons within the corticospinal tract to appropriately activate muscles. This possibility is later confirmed in L6 by information that students obtain from the Online Mendelian Inheritance in Man (OMIM) database.		
**4**	**Exploring molecular genetics (foundational)**	**v.2**
In L1 students are likely to formulate questions about the involvement of genes in the disorder. This four-part lesson engages students in a foundational exploration of chromosomes, DNA, and the role that genes play in specifying the amino acid sequence, structure, and function of proteins used by cells to carry out essential life functions. Understanding this foundational information is required for students to later analyze, interpret, and connect evidence that links specific gene mutations and corresponding protein anomalies to the movement disorder.		
**5**	**Exploring medical genetics (foundational)**	**v.3**
In L1, students are also likely to formulate questions about the disorder’s heritability. In this lesson, students explore select examples of dominant and recessive gene alleles and their role in the expression of a particular phenotype (trait). They also explore the use of Punnett squares to predict the probability that offspring will inherit a phenotype (trait) from their parents, and the use of pedigree charts to show actual patterns of inheritance through multiple generations of a family. During this lesson, students also carry out an investigation to determine if the movement disorder under investigation results from a heritable mutation and to deduce the pattern of inheritance (e.g., autosomal dominant). In the final part of this lesson, students use a web-based application within the Human Phenotype Ontology database (Phenomizer) to diagnose the movement disorder. To perform the diagnosis, students indicate the disorder’s pattern of inheritance (obtained through pedigree analysis) and enter clinical features (symptoms) presented by affected patients (students obtain this information from semi-fictitious case reports of family members). The diagnosis report generated by the Phenomizer app not only includes the name of the disorder, but the name of genes linked to the disorder (which are hyperlinked to data records in the OMIM database). The role of these genes in the movement disorder will be the focus of student exploration in subsequent lessons.		
**6**	**Exploring genes and gene products linked to the movement disorder**	**v.4**
In this lesson, students navigate the OMIM database to identify four genes linked to the movement disorder. This finding connects to a key discovery made in L4 and L5 (i.e., that the movement disorder is genetically heritable). By analyzing data records contained in the database, students discover that the proteins encoded by two of these four genes (*NTN1* and *DCC*) physically interact during a key phase of nervous system development (axon pathfinding). The information that students encounter in OMIM data records also builds upon a key discovery made in L5, namely that the movement disorder results from a failure of axons within the corticospinal tract to appropriately cross the midline during nervous system development (abnormal corticospinal tract decussation). The next several lessons are designed to help students understand how physical interactions between Netrin-1 (encoded by the *NTN1* gene) and DCC proteins mediate midline crossing.		
**7**	**Exploring central nervous system (CNS) development (foundational)**	**v.4**
In L6, students identified two genes (*NTN1* and *DCC*) linked to the movement disorder. They also determined that the corresponding proteins (Netrin-1 and DCC) interact and play important roles in axon pathfinding, a key phase of nervous system development. In L7A and L7B, students explore the preceding phases of nervous system development (neurogenesis, cell fate specification) and examine models of axon pathfinding, the process by which differentiated neurons locate target cells with which they will ultimately establish functional connections (synapses). As part of this exploration, students examine the architecture of the axon and its terminal growth cone, neuronal structures that play key roles in axon pathfinding. They also analyze models that highlight how growth cones display cell surface receptors that are capable of recognizing and steering the axon in response to secreted navigational cues distributed within the developing nervous system.		
**8**	**Examining the role of genes in CNS development (model organisms and systems)**	**v.5**
In this lesson, students explore the concept of a model organism/system and the conservation of gene function across animal phyla. In L8A, students use the OpenWorm 3D modeling platform to examine and characterize the trajectories of wild-type *C. elegans* axons and make general comparisons to the pathway taken by the axons of upper (cortical) motor neurons that form the corticospinal tract in humans (e.g., axons in both systems cross the midline and travel significant distances before making contact with contralateral target cells). Students then examine how mutations in *Unc-6* (the *NTN1* ortholog) and *Unc-40* (the *DCC* ortholog) affect the pathways taken by axons that cross either the dorsal or ventral midline of the *C. elegans* body axis. In L8B, students turn their focus to a vertebrate system and evaluate the trajectories of hindbrain neurons in mice harboring mutations that affect the regional expression of *NTN1* gene.		
**9**	**Examining the role of genes in CNS development (human studies)**	**v.5**
In the previous lesson, students explored the trajectories of axons in model organisms (*C. elegans* and mouse) that harbor mutations in either the *NTN1* or *DCC* genes. In both organisms, mutations in either of these genes result in a failure of axons to cross the midline and project on the opposite (contralateral) side of the body axis (abnormal decussation). This observation connects to data encountered in L5 and L6. In L9A, students compare and evaluate diffusion tensor imaging (DTI) tractography data obtained from normal human subjects and individuals affected by the movement disorder. They discover that mutations in either *NTN1* or *DCC* result in a partial failure of axons within the corticospinal tract to cross the CNS midline at the level of the hindbrain (abnormal corticospinal tract decussation; see Figure 1). In L9B and L9C, students use online informatics tools and databases, most notably NCBI ClinVar, to examine *NTN1* and *DCC* gene variations (mutations) and determine their impact on the amino acid sequence of the corresponding proteins. Students also use the Simple Modular Architecture Research Tool to explore how mutations impair the ability of Netrin-1 and DCC proteins to physically interact with one another and perform a role in guiding upper (cortical) motor neurons across the hindbrain midline.		
**10**	**Completing an explanatory model for the movement disorder**	**v.6**
By this point in the NeuroLab storyline, students will have implicated defects in the DCC receptor or the Netrin-1 guidance cue as the primary cause of the movement disorder. In this first half of this concluding lesson, students use Brain Explorer 2 (a companion application of the Allen Developing Mouse Brain Atlas) to examine the spatial distribution of NTN1 and DCC mRNA in the developing mouse embryo and to evaluate the extent to which this expression data is consistent with the relevant components of their emerging explanatory model. In the second half of this lesson, students receive guidance on evaluating their model for the presence of different components (e.g., behavioral, anatomical, developmental, etc.) and are invited to discuss and provide explanations for any unresolved questions. The experience concludes by inviting students to propose a treatment for CMM based on their current understanding of the disorder.		

**Table 6.** NGSS performance expectations (PEs) addressed by the NeuroLab storyline unit.

HS-LS1–1	Construct an explanation based on evidence for how the structure of DNA determines the structure of proteins that carry out the essential functions of life through systems of specialized cells
HS-LS1–2	Develop and use a model to illustrate the hierarchical organization of interacting systems that provide specific functions within multicellular organisms.
HS-LS1–4	Use a model to illustrate the role of cellular division (mitosis) and differentiation in producing and maintaining complex organisms.
HS-LS3–1	Ask questions to clarify relationships about the role of DNA and chromosomes in coding the instructions for characteristic traits passed from parents to offspring.
HS-LS3–2	Make and defend a claim based on evidence that inheritable genetic variations may result from: (1) new genetic combinations through meiosis, (2) viable errors occurring during replication, and/or (3) mutations caused by environmental factors.

Note: Student performance demonstrations may cross the assessment boundaries for several PEs displayed in this table (refer to S2 for additional details). However, as teacher co-designers noted, the learning objectives of courses in which the storyline is likely to be implemented may require students to demonstrate an understanding of ideas and concepts that exceed these assessment boundaries.

## ABBREVIATIONS

CMM: Congenital Mirror Movement Disorder
CNS: Central Nervous System
ISE: Informal Science Experience
NGSS: Next Generation Science Standards
SEPA: Science Education Partnership Award
STEM+M: Science, Technology, Engineering, Mathematics, and Medicine
