# Supplementary material for: NeuroLab 2.0: An Alternative Storyline Design Approach for Translating a Research-Based Summer Experience into an Advanced STEM+M Curriculum Unit that Supports Three-Dimensional Teaching and Learning in the Classroom
Source: J STEM Outreach. Author manuscript; Available in PMC 2024 Jun 4. (PMC11149925; doi:10.15695/jstem/v7i1.03)
Supplement: S1 [file NIHMS1973000-supplement-S1.pdf]

Muscle activation was recorded by EMG from both hands while the patients performed the motor task

Patient 1

Contralateral (passive) hand
(non-dominant hand)

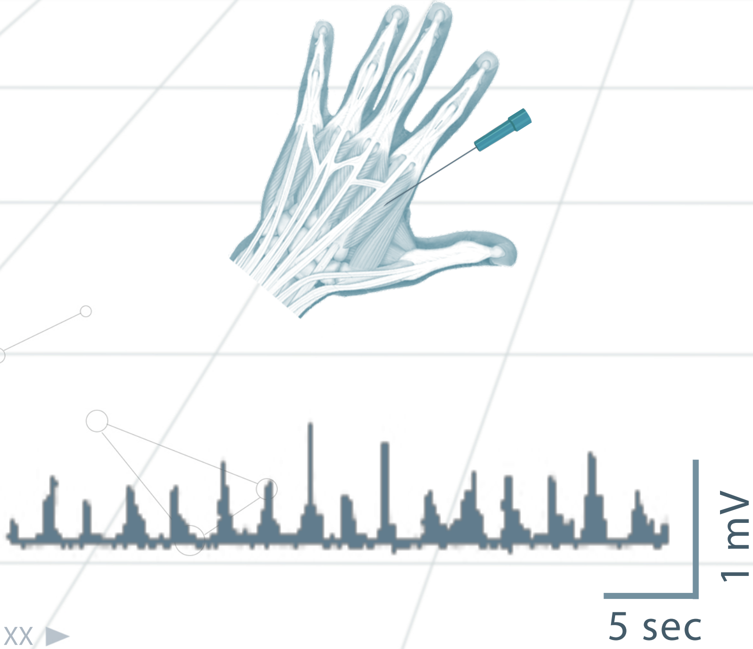

Motor task hand
(dominant hand)

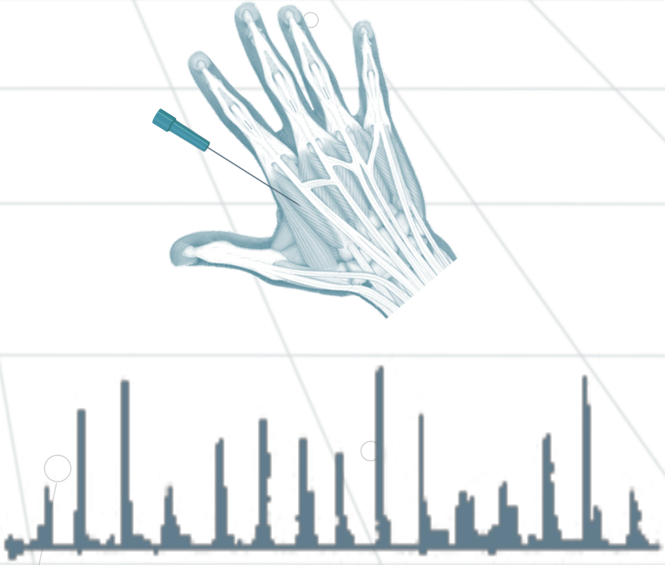

Adapted from Fosano et al., 2014

About how long did Patient 1 perform the clinical motor task?

Patient 1

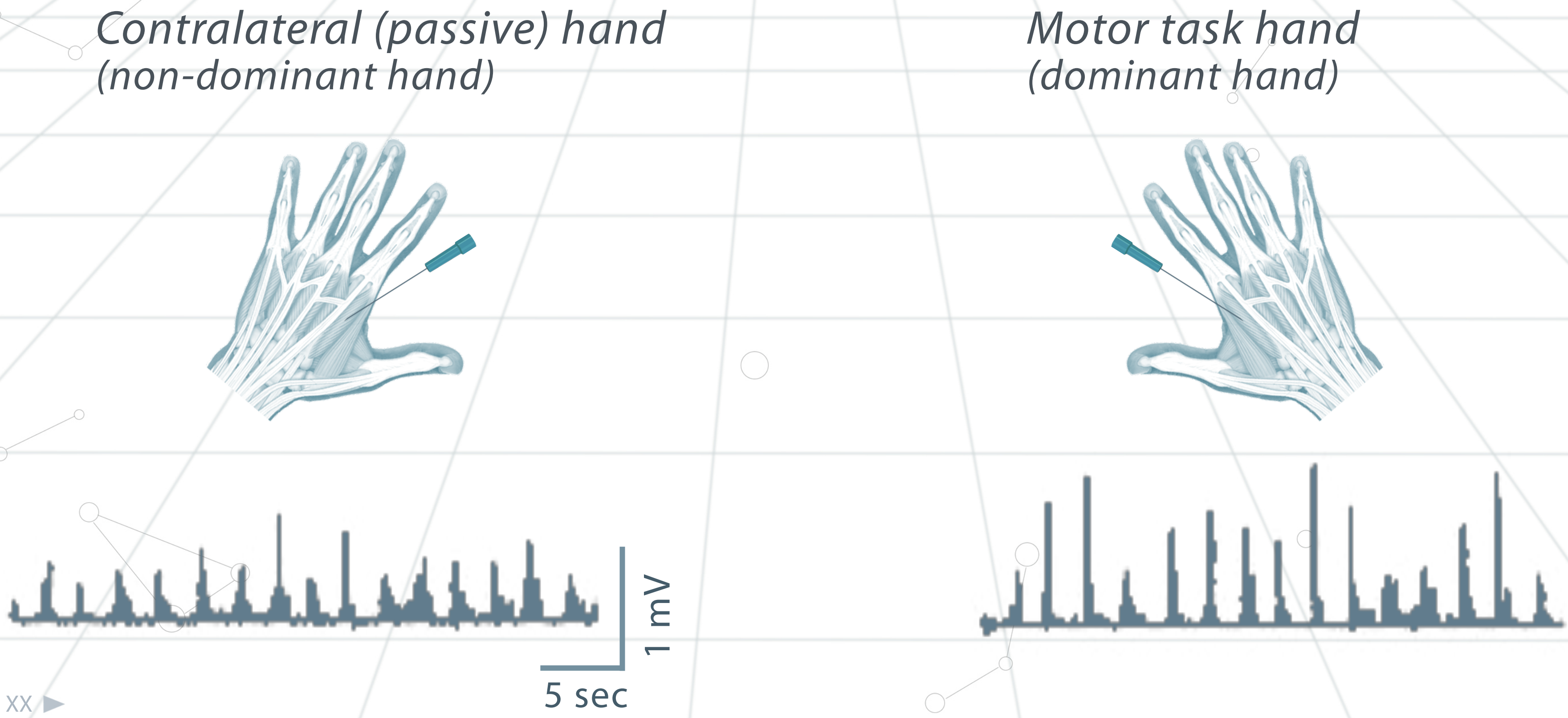

Adapted from Fosano et al., 2014

Patient 1 performed the clinical motor task for about 30 seconds

Patient 1

Contralateral (passive) hand
(non-dominant hand)

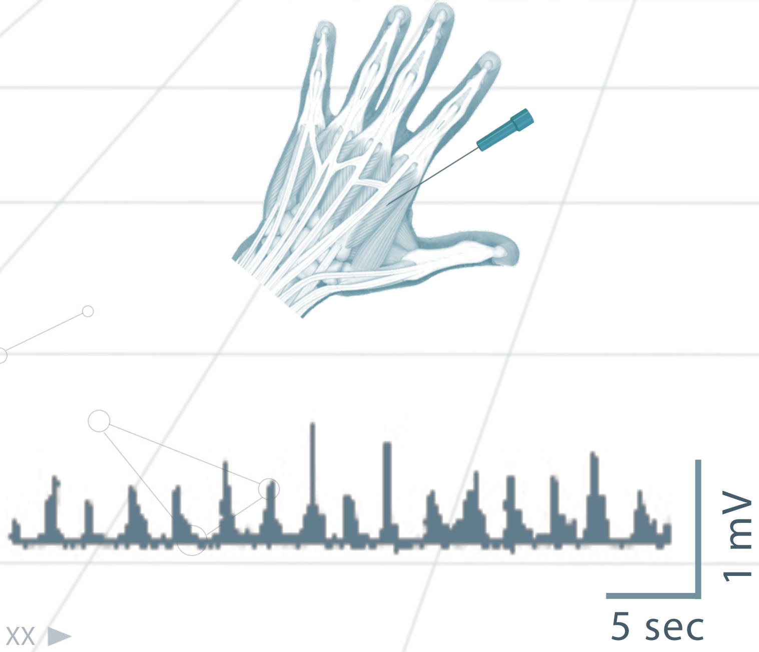

Motor task hand
(dominant hand)

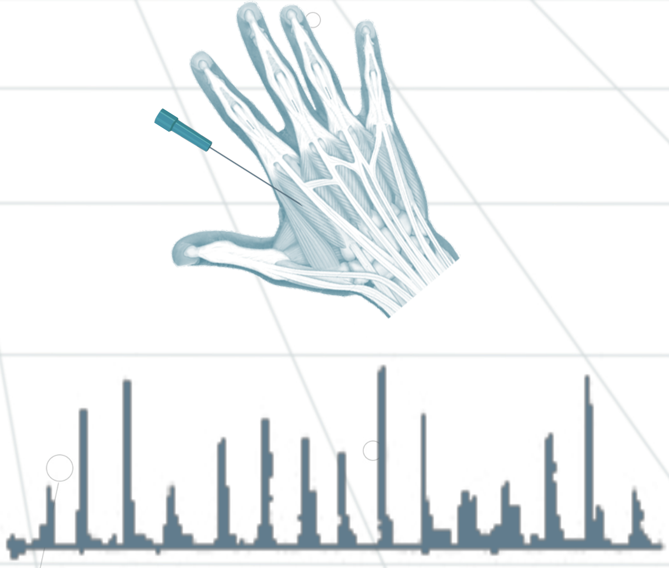

Adapted from Fosano et al., 2014

What do the peaks or spikes represent in the two graphs below?

Patient 1

Contralateral (passive) hand
(non-dominant hand)

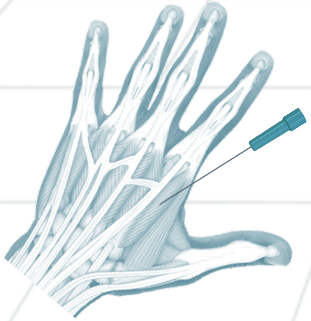

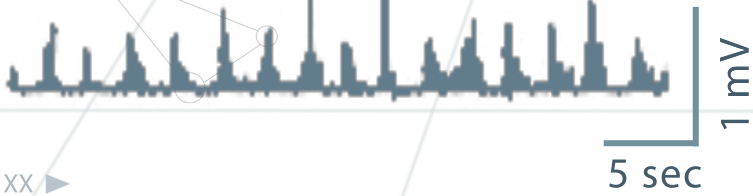

Motor task hand
(dominant hand)

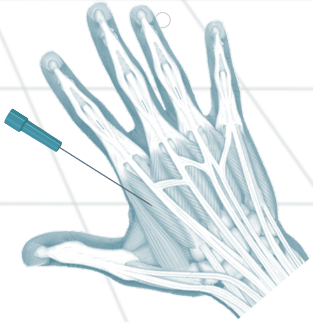

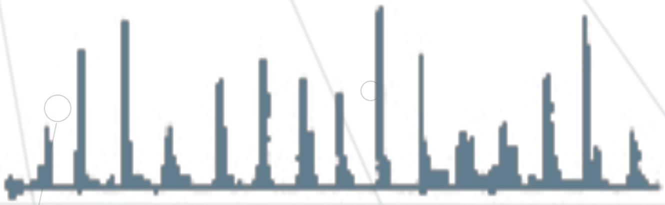

Adapted from Fosano et al., 2014

The spikes represent the level of FDI muscle activation

Patient 1

Contralateral (passive) hand
(non-dominant hand)

Motor task hand
(dominant hand)

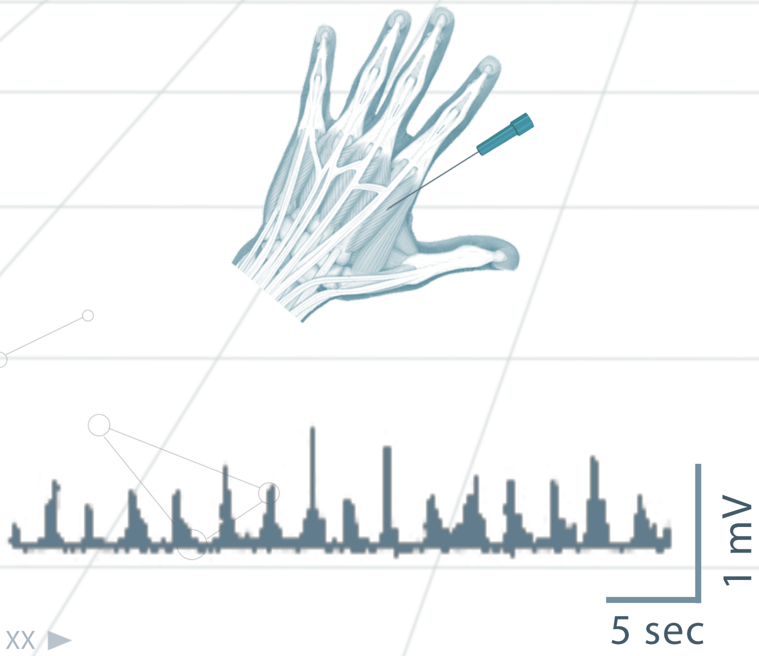

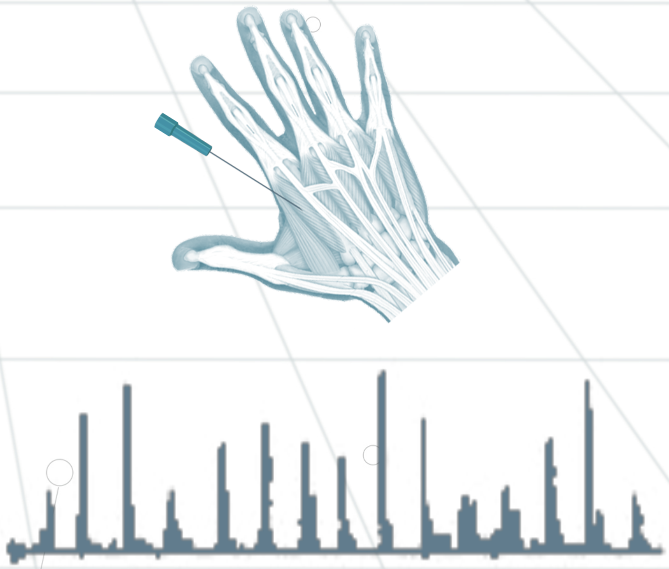

Adapted from Fosano et al., 2014

MotionLab

Spinal cord neurons on what side of the body should be activating FDI muscles in the task hand?

Patient 1

Contralateral (passive) hand
(non-dominant hand)

Motor task hand
(dominant hand)

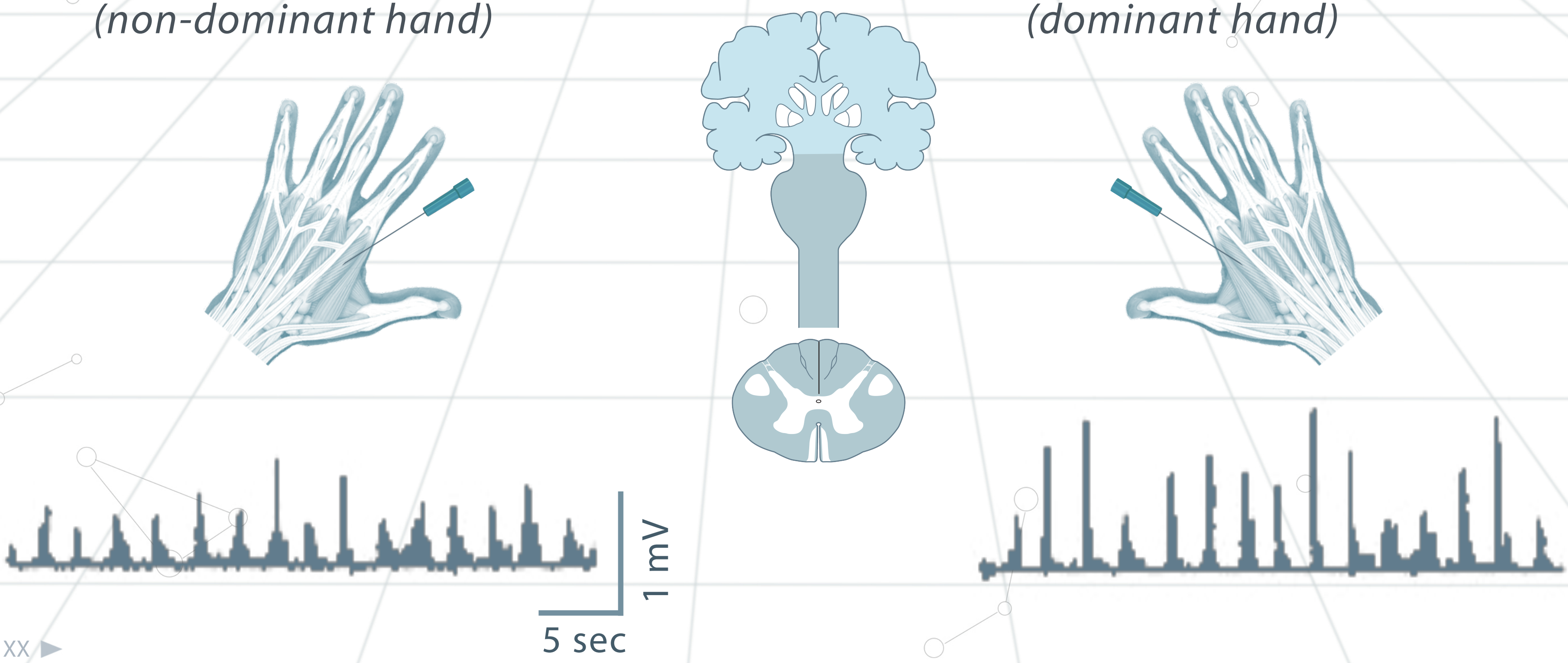

Adapted from Fosano et al., 2014

Cortical motor neurons on what side of the body should be activating FDI muscles in the task hand?

Patient 1

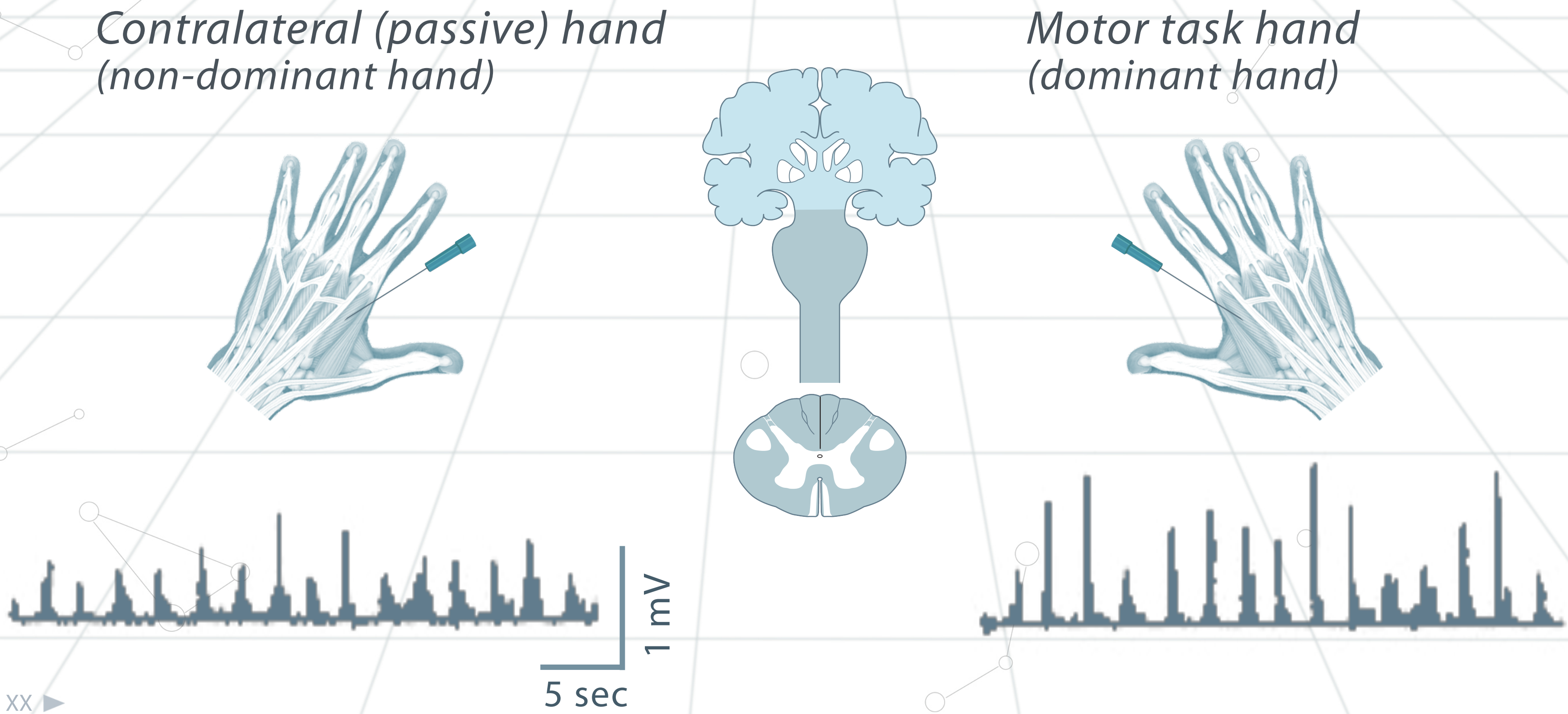

Adapted from Fosano et al., 2014

What does the gap or interval between peaks represent?

Patient 1

Contralateral (passive) hand
(non-dominant hand)

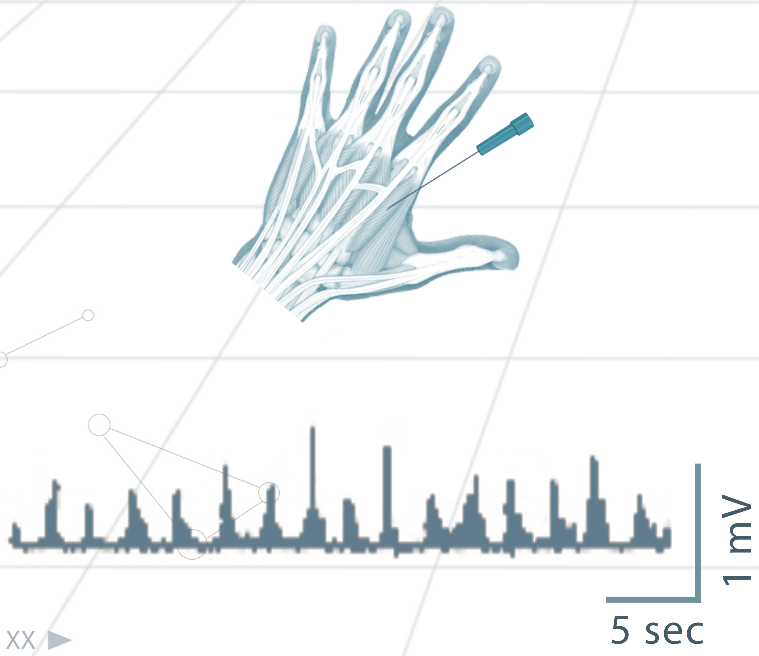

Motor task hand
(dominant hand)

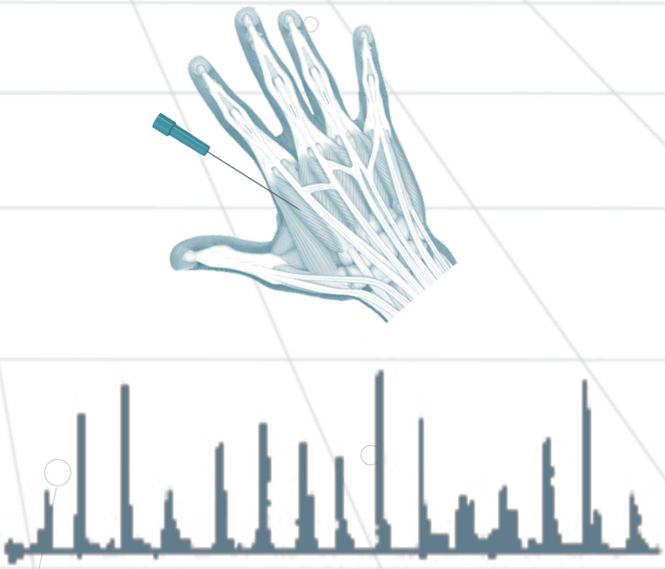

Adapted from Fosano et al., 2014

The interval between peaks represents the time between finger tapping commands

Patient 1

Contralateral (passive) hand
(non-dominant hand)

Motor task hand
(dominant hand)

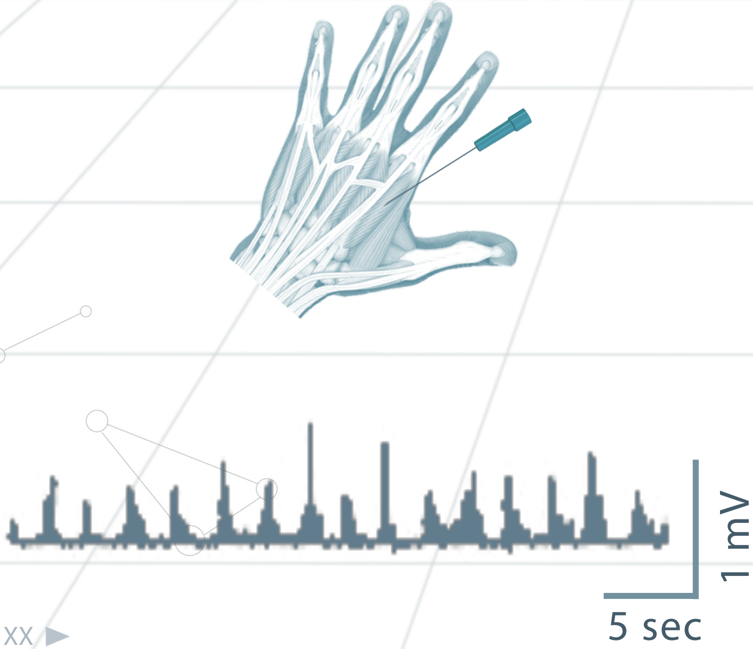

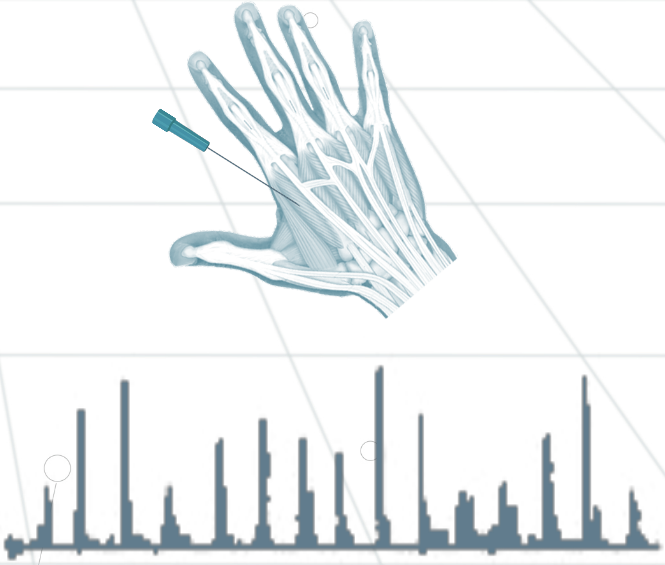

Adapted from Fosano et al., 2014

What does the height or amplitude of the peaks indicate in the graphs?

Patient 1

Contralateral (passive) hand
(non-dominant hand)

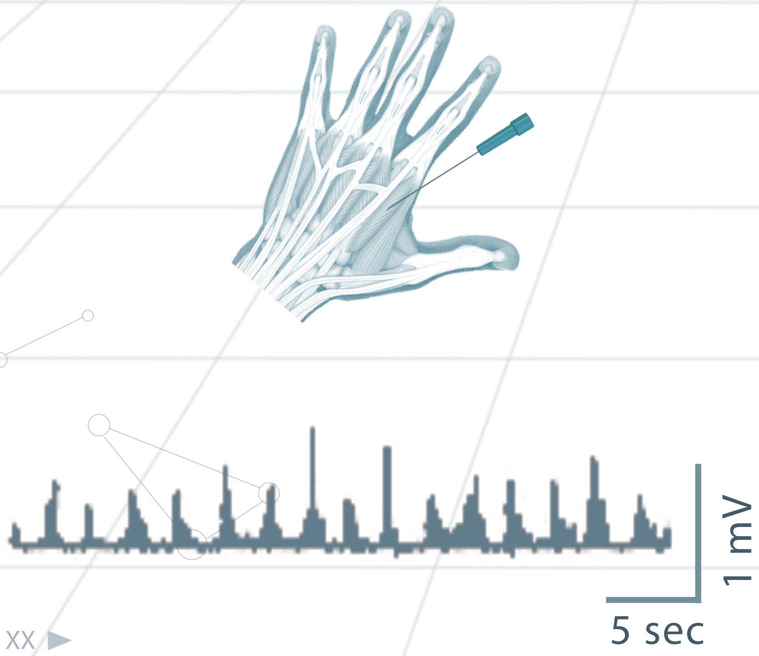

Motor task hand
(dominant hand)

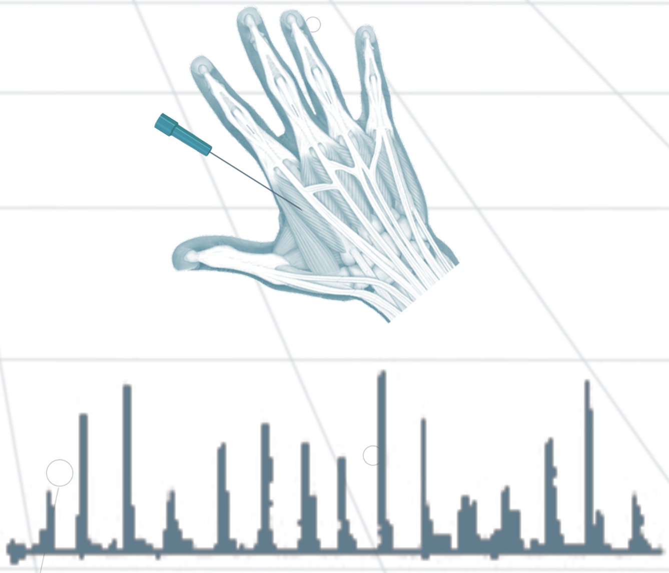

Adapted from Fosano et al., 2014

Is there a difference in the height or amplitude of the peaks generated by the task hand and the contralateral passive hand?

Patient 1

Contralateral (passive) hand
(non-dominant hand)

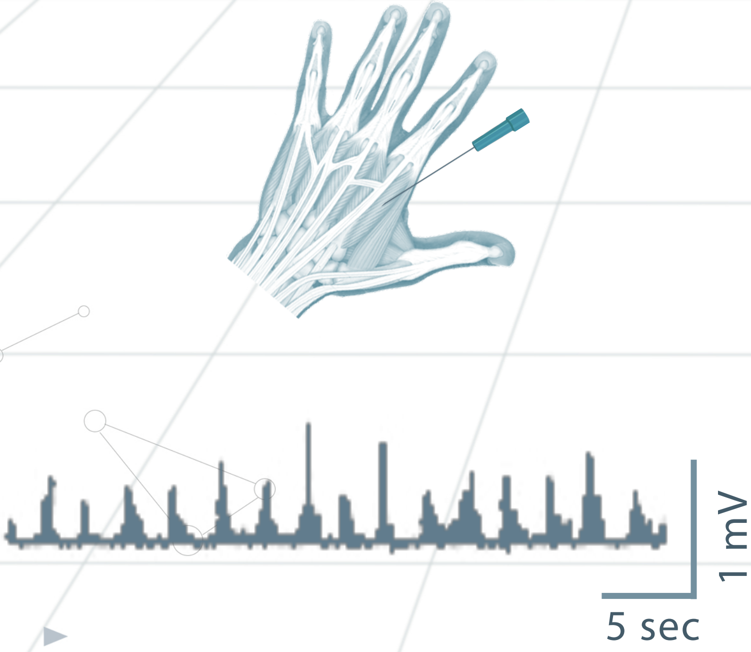

Motor task hand
(dominant hand)

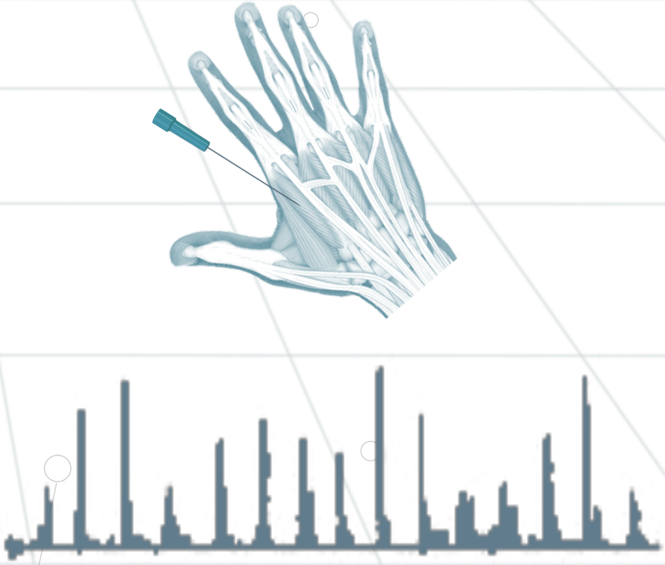

Adapted from Fosano et al., 2014

How does this difference compare (correlate) with what you observed in the videos of affected patients?

Patient 1

Contralateral (passive) hand
(non-dominant hand)

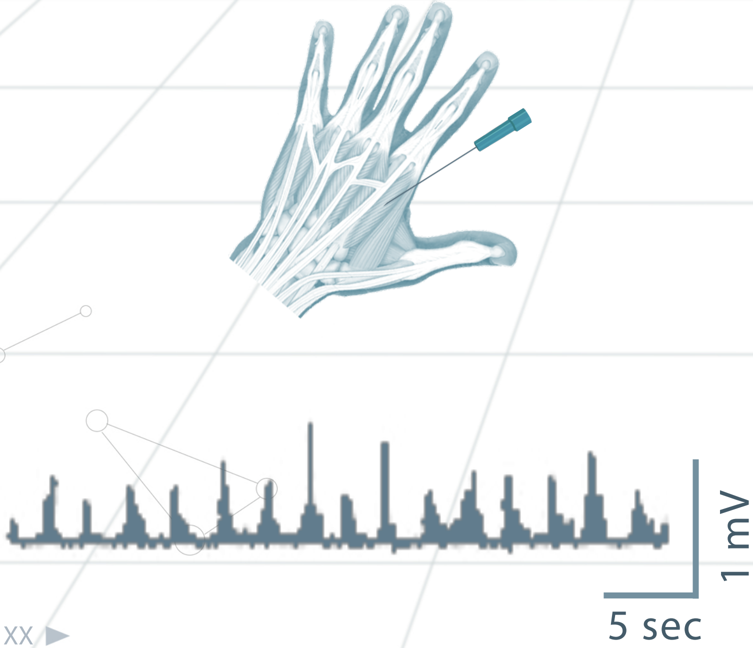

Motor task hand
(dominant hand)

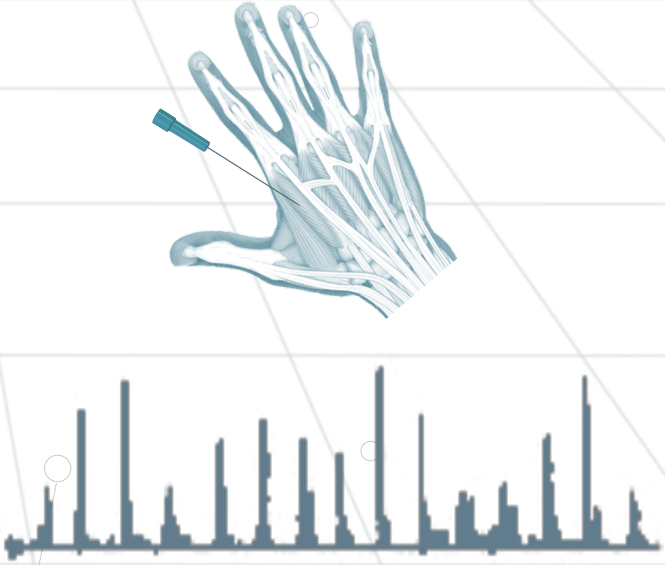

Adapted from Fosano et al., 2014

Based on this data, would this patient show a higher or lower degree of hand mirroring by visual observation?

Patient 1

Contralateral (passive) hand
(non-dominant hand)

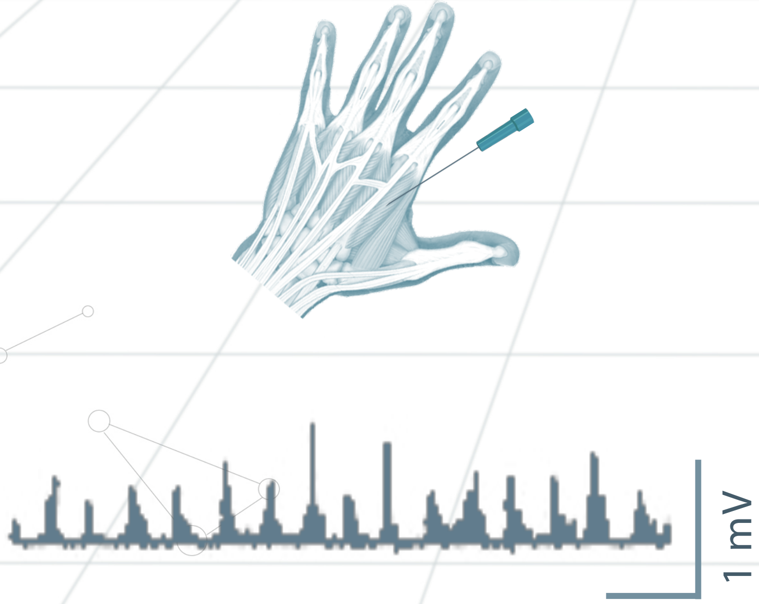

Motor task hand
(dominant hand)

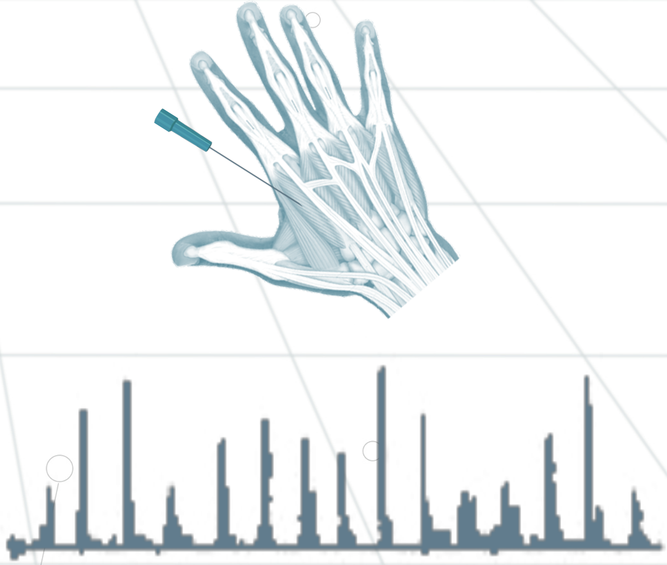

Adapted from Fosano et al., 2014
