# Supplementary material for: NeuroLab 2.0: An Alternative Storyline Design Approach for Translating a Research-Based Summer Experience into an Advanced STEM+M Curriculum Unit that Supports Three-Dimensional Teaching and Learning in the Classroom
Source: J STEM Outreach. Author manuscript; Available in PMC 2024 Jun 4. (PMC11149925; doi:10.15695/jstem/v7i1.03)
Supplement: S2 [file NIHMS1973000-supplement-S2.pdf]

The MRMV1 data record contains a <Phenotypic Series> tab

157600

MIRROR MOVEMENTS 1; MRMV1

Alternative titles; symbols

MIRROR MOVEMENTS 1 AND/OR AGENESIS OF THE CORPUS CALLOSUM
MIRROR MOVEMENTS, CONGENITAL
BIMANUAL SYNERGIA

Phenotype-Gene Relationships

Location	Phenotype	Phenotype MIM number	Inheritance	Phenotype mapping key	Gene/Locus	Gene/Locus MIM number
18q21.2	Mirror movements 1 and/or agenesis of the corpus callosum	157600	AD	3	DCC	120470

Clinical Synopsis | Phenotypic Series | PheneGene Graphics

NeuroLab

A phenotypic series is a group of similar or identical phenotypes that arise from different gene mutations

\# 157600

MIRROR MOVEMENTS 1; MRMV1

Alternative titles; symbols

MIRROR MOVEMENTS 1 AND/OR AGENESIS OF THE CORPUS CALLOSUM
MIRROR MOVEMENTS, CONGENITAL
BIMANUAL SYNERGIA

Phenotype-Gene Relationships

Location	Phenotype	Phenotype MIM number	Inheritance	Phenotype mapping key	Gene/Locus	Gene/Locus MIM number
18q21.2	Mirror movements 1 and/or agenesis of the corpus callosum	157600	AD	3	DCC	120470

Clinical Synopsis | Phenotypic Series | PheneGene Graphics

Click the tab to view a table that summarizes the phenotypic series

\# 157600

MIRROR MOVEMENTS 1; MRMV1

Alternative titles; symbols

MIRROR MOVEMENTS 1 AND/OR AGENESIS OF THE CORPUS CALLOSUM
MIRROR MOVEMENTS, CONGENITAL
BIMANUAL SYNERGIA

Phenotype-Gene Relationships

Location	Phenotype	Phenotype MIM number	Inheritance	Phenotype mapping key	Gene/Locus	Gene/Locus MIM number
18q21.2	Mirror movements 1 and/or agenesis of the corpus callosum	157600	AD	3	DCC	120470

Clinical Synopsis ▾ | Phenotypic Series ▾ | PheneGene Graphics ▾ | ?

NeuroLab

How many similar phenotypes are displayed in the table? Which genes are associated with the phenotypes?

Phenotypic Series - PS157600

Mirror movements - PS157600 - 4 Entries

View corresponding clinical synopses as a table

Location	Phenotype	Inheritance	Phenotype mapping key	Phenotype MIM number	Gene/Locus	Gene/Locus MIM number
15q15.1	Mirror movements 2	AD	3	614508	RAD51	179617
17p13.1	Mirror movements 4	AD	3	618264	NTN1	601614
18q21.2	Mirror movements 1 and/or agenesis of the corpus callosum	AD	3	157600	DCC	120470
22q13.1	?Mirror movements 3	AR	3	616059	DNAL4	610565

View corresponding clinical synopses as a table

PheneGene Graphics

NeuroLab

The table displays 4 phenotypes corresponding to 4 separate genes

Phenotypic Series - PS157600

Mirror movements - PS157600 - 4 Entries

View corresponding clinical synopses as a table

Location	Phenotype	Inheritance	Phenotype mapping key	Phenotype MIM number	Gene/Locus	Gene/Locus MIM number
15q15.1	Mirror movements 2	AD	3	614508	RAD51	179617
17p13.1	Mirror movements 4	AD	3	618264	NTN1	601614
18q21.2	Mirror movements 1 and/or agenesis of the corpus callosum	AD	3	157600	DCC	120470
22q13.1	?Mirror movements 3	AR	3	616059	DNAL4	610565

View corresponding clinical synopses as a table

PheneGene Graphics

NeuroLab

Click the link at the bottom right to open a table that summarizes the clinical findings for each phenotype

Phenotypic Series – PS157600

Mirror movements – PS157600 – 4 Entries

View corresponding clinical synopses as a table

Location	Phenotype	Inheritance	Phenotype mapping key	Phenotype MIM number	Gene/Locus	Gene/Locus MIM number
15q15.1	Mirror movements 2	AD	3	614508	RAD51	179617
17p13.1	Mirror movements 4	AD	3	618264	NTN1	601614
18q21.2	Mirror movements 1 and/or agenesis of the corpus callosum	AD	3	157600	DCC	120470
22q13.1	?Mirror movements 3	AR	3	616059	DNAL4	610565

View corresponding clinical synopses as a table

PheneGene Graphics

NeuroLab

How would you describe the similarity of neurologic findings displayed in the shaded row of this table?

**NUMBER**	# 614508	# 618264	# 157600	# 616059
**TITLE**	MIRROR MOVEMENTS 2; MRMV2	MIRROR MOVEMENTS 4; MRMV4	MIRROR MOVEMENTS 1; MRMV1	MIRROR MOVEMENTS 3; MRMV3
**INHERITANCE** (in 4/4)	- Autosomal dominant	- Autosomal dominant	- Autosomal dominant	- Autosomal recessive
**ABDOMEN** (in 1/4) ▼		*Gastrointestinal*<br>- Irritable bowel syndrome, constipation-dominant (in some patients)		
**MUSCLE, SOFT TISSUES** (in 2/4) ▼	- Pain or cramping during sustained manual activity		- Pain or cramping during sustained manual activity	
**NEUROLOGIC** (in 4/4) ▼	*Central Nervous System*<br>- Mirror movements, involuntary, usually of the upper limb and hand<br>- Difficulties in fine bimanual activities<br>- Writing fatigability<br>- Abnormal corticospinal tract decussation	*Central Nervous System*<br>- Mirror movements, involuntary, usually of the upper limb and hand<br>- Difficulties in fine bimanual activities<br>- Abnormal corticospinal tract decussation	*Central Nervous System*<br>- Intellectual disability, mild (in some patients)<br>- Mirror movements, involuntary, usually of the upper limb and hand<br>- Difficulties in fine bimanual activities<br>- Writing fatigability<br>- Abnormal corticospinal tract decussation<br>- Agenesis of the corpus callosum (in some patients)<br>- Absence of hippocampal commissure	*Central Nervous System*<br>- Mirror movements, involuntary, affecting the hand and fingers<br>- Difficulties in fine bimanual activities
**MISCELLANEOUS** (in 4/4) ▼	- Onset in infancy or early childhood<br>- Disorder usually remains stable over time<br>- Incomplete penetrance (50%)	- Onset in early childhood<br>- Three unrelated families have been reported (last curated December 2018)	- Onset in infancy or early childhood<br>- Disorder usually remains stable over time<br>- Highly variable phenotype<br>- Incomplete penetrance	- Onset in infancy or early childhood<br>- One consanguineous Pakistani family has been reported (last curated October 2014)
**MOLECULAR BASIS** (in 4/4) ▼	- Caused by mutation in the homolog of S. cerevisiae RAD51 gene (RAD51, 179617.0003)	- Caused by mutation in the netrin 1 gene (NTN1, 601614.0001)	- Caused by mutation in the deleted in colorectal carcinoma gene (DCC, 120470.0001)	- Caused by mutation in the dynein, axonemal, light chain 4 gene (DNAL4, 610565.0001)

NeuroLab

Which phenotypes are associated with involuntary mirror movements?

**NUMBER**	# 614508	# 618264	# 157600	# 616059
**TITLE**	MIRROR MOVEMENTS 2; MRMV2	MIRROR MOVEMENTS 4; MRMV4	MIRROR MOVEMENTS 1; MRMV1	MIRROR MOVEMENTS 3; MRMV3
**INHERITANCE** (in 4/4)	- Autosomal dominant	- Autosomal dominant	- Autosomal dominant	- Autosomal recessive
**ABDOMEN** (in 1/4) ▼		*Gastrointestinal*<br>- Irritable bowel syndrome, constipation-dominant (in some patients)		
**MUSCLE, SOFT TISSUES** (in 2/4) ▼	- Pain or cramping during sustained manual activity		- Pain or cramping during sustained manual activity	
**NEUROLOGIC** (in 4/4) ▼	*Central Nervous System*<br>- Mirror movements, involuntary, usually of the upper limb and hand<br>- Difficulties in fine bimanual activities<br>- Writing fatigability<br>- Abnormal corticospinal tract decussation	*Central Nervous System*<br>- Mirror movements, involuntary, usually of the upper limb and hand<br>- Difficulties in fine bimanual activities<br>- Abnormal corticospinal tract decussation	*Central Nervous System*<br>- Intellectual disability, mild (in some patients)<br>- Mirror movements, involuntary, usually of the upper limb and hand<br>- Difficulties in fine bimanual activities<br>- Writing fatigability<br>- Abnormal corticospinal tract decussation<br>- Agenesis of the corpus callosum (in some patients)<br>- Absence of hippocampal commissure	*Central Nervous System*<br>- Mirror movements, involuntary, affecting the hand and fingers<br>- Difficulties in fine bimanual activities
**MISCELLANEOUS** (in 4/4) ▼	- Onset in infancy or early childhood<br>- Disorder usually remains stable over time<br>- Incomplete penetrance (50%)	- Onset in early childhood<br>- Three unrelated families have been reported (last curated December 2018)	- Onset in infancy or early childhood<br>- Disorder usually remains stable over time<br>- Highly variable phenotype<br>- Incomplete penetrance	- Onset in infancy or early childhood<br>- One consanguineous Pakistani family has been reported (last curated October 2014)
**MOLECULAR BASIS** (in 4/4) ▼	- Caused by mutation in the homolog of S. cerevisiae RAD51 gene (RAD51, 179617.0003)	- Caused by mutation in the netrin 1 gene (NTN1, 601614.0001)	- Caused by mutation in the deleted in colorectal carcinoma gene (DCC, 120470.0001)	- Caused by mutation in the dynein, axonemal, light chain 4 gene (DNAL4, 610565.0001)

Which phenotypes are associated with difficulties in fine bimanual activities?

**NUMBER**	# 614508	# 618264	# 157600	# 616059
**TITLE**	MIRROR MOVEMENTS 2; MRMV2	MIRROR MOVEMENTS 4; MRMV4	MIRROR MOVEMENTS 1; MRMV1	MIRROR MOVEMENTS 3; MRMV3
**INHERITANCE** (in 4/4)	- Autosomal dominant	- Autosomal dominant	- Autosomal dominant	- Autosomal recessive
**ABDOMEN** (in 1/4) ▼		*Gastrointestinal*<br>- Irritable bowel syndrome, constipation-dominant (in some patients)		
**MUSCLE, SOFT TISSUES** (in 2/4) ▼	- Pain or cramping during sustained manual activity		- Pain or cramping during sustained manual activity	
**NEUROLOGIC** (in 4/4) ▼	*Central Nervous System*<br>- Mirror movements, involuntary, usually of the upper limb and hand<br>- Difficulties in fine bimanual activities<br>- Writing fatigability<br>- Abnormal corticospinal tract decussation	*Central Nervous System*<br>- Mirror movements, involuntary, usually of the upper limb and hand<br>- Difficulties in fine bimanual activities<br>- Abnormal corticospinal tract decussation	*Central Nervous System*<br>- Intellectual disability, mild (in some patients)<br>- Mirror movements, involuntary, usually of the upper limb and hand<br>- Difficulties in fine bimanual activities<br>- Writing fatigability<br>- Abnormal corticospinal tract decussation<br>- Agenesis of the corpus callosum (in some patients)<br>- Absence of hippocampal commissure	*Central Nervous System*<br>- Mirror movements, involuntary, affecting the hand and fingers<br>- Difficulties in fine bimanual activities
**MISCELLANEOUS** (in 4/4) ▼	- Onset in infancy or early childhood<br>- Disorder usually remains stable over time<br>- Incomplete penetrance (50%)	- Onset in early childhood<br>- Three unrelated families have been reported (last curated December 2018)	- Onset in infancy or early childhood<br>- Disorder usually remains stable over time<br>- Highly variable phenotype<br>- Incomplete penetrance	- Onset in infancy or early childhood<br>- One consanguineous Pakistani family has been reported (last curated October 2014)
**MOLECULAR BASIS** (in 4/4) ▼	- Caused by mutation in the homolog of S. cerevisiae RAD51 gene (RAD51, 179617.0003)	- Caused by mutation in the netrin 1 gene (NTN1, 601614.0001)	- Caused by mutation in the deleted in colorectal carcinoma gene (DCC, 120470.0001)	- Caused by mutation in the dynein, axonemal, light chain 4 gene (DNAL4, 610565.0001)

NeuroLab

Which phenotypes are associated with abnormal corticospinal tract crossing (decussation)?

NUMBER	# 614508	# 618264	# 157600	# 616059
TITLE	MIRROR MOVEMENTS 2; MRMV2	MIRROR MOVEMENTS 4; MRMV4	MIRROR MOVEMENTS 1; MRMV1	MIRROR MOVEMENTS 3; MRMV3
INHERITANCE (in 4/4)	- Autosomal dominant	- Autosomal dominant	- Autosomal dominant	- Autosomal recessive
ABDOMEN (in 1/4) ▼		*Gastrointestinal*<br>- Irritable bowel syndrome, constipation-dominant (in some patients)		
MUSCLE, SOFT TISSUES (in 2/4) ▼	- Pain or cramping during sustained manual activity		- Pain or cramping during sustained manual activity	
NEUROLOGIC (in 4/4) ▼	*Central Nervous System*<br>- Mirror movements, involuntary, usually of the upper limb and hand<br>- Difficulties in fine bimanual activities<br>- Writing fatigability<br>- Abnormal corticospinal tract decussation	*Central Nervous System*<br>- Mirror movements, involuntary, usually of the upper limb and hand<br>- Difficulties in fine bimanual activities<br>- Abnormal corticospinal tract decussation	*Central Nervous System*<br>- Intellectual disability, mild (in some patients)<br>- Mirror movements, involuntary, usually of the upper limb and hand<br>- Difficulties in fine bimanual activities<br>- Writing fatigability<br>- Abnormal corticospinal tract decussation<br>- Agenesis of the corpus callosum (in some patients)<br>- Absence of hippocampal commissure	*Central Nervous System*<br>- Mirror movements, involuntary, affecting the hand and fingers<br>- Difficulties in fine bimanual activities
MISCELLANEOUS (in 4/4) ▼	- Onset in infancy or early childhood<br>- Disorder usually remains stable over time<br>- Incomplete penetrance (50%)	- Onset in early childhood<br>- Three unrelated families have been reported (last curated December 2018)	- Onset in infancy or early childhood<br>- Disorder usually remains stable over time<br>- Highly variable phenotype<br>- Incomplete penetrance	- Onset in infancy or early childhood<br>- One consanguineous Pakistani family has been reported (last curated October 2014)
MOLECULAR BASIS (in 4/4) ▼	- Caused by mutation in the homolog of S. cerevisiae RAD51 gene (RAD51, 179617.0003)	- Caused by mutation in the netrin 1 gene (NTN1, 601614.0001)	- Caused by mutation in the deleted in colorectal carcinoma gene (DCC, 120470.0001)	- Caused by mutation in the dynein, axonemal, light chain 4 gene (DNAL4, 610565.0001)

NeuroLab

Which phenotypes are associated with abnormal corticospinal tract crossing?

NUMBER	# 614508	# 618264	# 157600	# 616059
TITLE	MIRROR MOVEMENTS 2; MRMV2	MIRROR MOVEMENTS 4; MRMV4	MIRROR MOVEMENTS 1; MRMV1	MIRROR MOVEMENTS 3; MRMV3
INHERITANCE (in 4/4)	- Autosomal dominant	- Autosomal dominant	- Autosomal dominant	- Autosomal recessive
ABDOMEN (in 1/4) ▼		*Gastrointestinal*<br>- Irritable bowel syndrome, constipation-dominant (in some patients)		
MUSCLE, SOFT TISSUES (in 2/4) ▼	- Pain or cramping during sustained manual activity		- Pain or cramping during sustained manual activity	
NEUROLOGIC (in 4/4) ▼	*Central Nervous System*<br>- Mirror movements, involuntary, usually of the upper limb and hand<br>- Difficulties in fine bimanual activities<br>- Writing fatigability<br>- Abnormal corticospinal tract decussation	*Central Nervous System*<br>- Mirror movements, involuntary, usually of the upper limb and hand<br>- Difficulties in fine bimanual activities<br>- Abnormal corticospinal tract decussation	*Central Nervous System*<br>- Intellectual disability, mild (in some patients)<br>- Mirror movements, involuntary, usually of the upper limb and hand<br>- Difficulties in fine bimanual activities<br>- Writing fatigability<br>- Abnormal corticospinal tract decussation<br>- Agenesis of the corpus callosum (in some patients)<br>- Absence of hippocampal commissure	*Central Nervous System*<br>- Mirror movements, involuntary, affecting the hand and fingers<br>- Difficulties in fine bimanual activities
MISCELLANEOUS (in 4/4) ▼	- Onset in infancy or early childhood<br>- Disorder usually remains stable over time<br>- Incomplete penetrance (50%)	- Onset in early childhood<br>- Three unrelated families have been reported (last curated December 2018)	- Onset in infancy or early childhood<br>- Disorder usually remains stable over time<br>- Highly variable phenotype<br>- Incomplete penetrance	- Onset in infancy or early childhood<br>- One consanguineous Pakistani family has been reported (last curated October 2014)
MOLECULAR BASIS (in 4/4) ▼	- Caused by mutation in the homolog of S. cerevisiae RAD51 gene (RAD51, 179617.0003)	- Caused by mutation in the netrin 1 gene (NTN1, 601614.0001)	- Caused by mutation in the deleted in colorectal carcinoma gene (DCC, 120470.0001)	- Caused by mutation in the dynein, axonemal, light chain 4 gene (DNAL4, 610565.0001)

NeuroLab

Explain how abnormal corticospinal tract decussation is relevant to the EMG, TMS, and fMRI data that you analyzed

NUMBER	# 614508	# 618264	# 157600	# 616059
TITLE	MIRROR MOVEMENTS 2; MRMV2	MIRROR MOVEMENTS 4; MRMV4	MIRROR MOVEMENTS 1; MRMV1	MIRROR MOVEMENTS 3; MRMV3
INHERITANCE (in 4/4)	- Autosomal dominant	- Autosomal dominant	- Autosomal dominant	- Autosomal recessive
ABDOMEN (in 1/4) ▼		*Gastrointestinal*<br>- Irritable bowel syndrome, constipation-dominant (in some patients)		
MUSCLE, SOFT TISSUES (in 2/4) ▼	- Pain or cramping during sustained manual activity		- Pain or cramping during sustained manual activity	
NEUROLOGIC (in 4/4) ▼	*Central Nervous System*<br>- Mirror movements, involuntary, usually of the upper limb and hand<br>- Difficulties in fine bimanual activities<br>- Writing fatigability<br>- Abnormal corticospinal tract decussation	*Central Nervous System*<br>- Mirror movements, involuntary, usually of the upper limb and hand<br>- Difficulties in fine bimanual activities<br>- Abnormal corticospinal tract decussation	*Central Nervous System*<br>- Intellectual disability, mild (in some patients)<br>- Mirror movements, involuntary, usually of the upper limb and hand<br>- Difficulties in fine bimanual activities<br>- Writing fatigability<br>- Abnormal corticospinal tract decussation<br>- Agenesis of the corpus callosum (in some patients)<br>- Absence of hippocampal commissure	*Central Nervous System*<br>- Mirror movements, involuntary, affecting the hand and fingers<br>- Difficulties in fine bimanual activities
MISCELLANEOUS (in 4/4) ▼	- Onset in infancy or early childhood<br>- Disorder usually remains stable over time<br>- Incomplete penetrance (50%)	- Onset in early childhood<br>- Three unrelated families have been reported (last curated December 2018)	- Onset in infancy or early childhood<br>- Disorder usually remains stable over time<br>- Highly variable phenotype<br>- Incomplete penetrance	- Onset in infancy or early childhood<br>- One consanguineous Pakistani family has been reported (last curated October 2014)
MOLECULAR BASIS (in 4/4) ▼	- Caused by mutation in the homolog of S. cerevisiae RAD51 gene (RAD51, 179617.0003)	- Caused by mutation in the netrin 1 gene (NTN1, 601614.0001)	- Caused by mutation in the deleted in colorectal carcinoma gene (DCC, 120470.0001)	- Caused by mutation in the dynein, axonemal, light chain 4 gene (DNAL4, 610565.0001)

NeuroLab

Explain how abnormal corticospinal tract decussation is relevant to your models?

NUMBER	# 614508	# 618264	# 157600	# 616059
TITLE	MIRROR MOVEMENTS 2; MRMV2	MIRROR MOVEMENTS 4; MRMV4	MIRROR MOVEMENTS 1; MRMV1	MIRROR MOVEMENTS 3; MRMV3
INHERITANCE (in 4/4)	- Autosomal dominant	- Autosomal dominant	- Autosomal dominant	- Autosomal recessive
ABDOMEN (in 1/4) ▼		*Gastrointestinal*<br>- Irritable bowel syndrome, constipation-dominant (in some patients)		
MUSCLE, SOFT TISSUES (in 2/4) ▼	- Pain or cramping during sustained manual activity		- Pain or cramping during sustained manual activity	
NEUROLOGIC (in 4/4) ▼	*Central Nervous System*<br>- Mirror movements, involuntary, usually of the upper limb and hand<br>- Difficulties in fine bimanual activities<br>- Writing fatigability<br>- Abnormal corticospinal tract decussation	*Central Nervous System*<br>- Mirror movements, involuntary, usually of the upper limb and hand<br>- Difficulties in fine bimanual activities<br>- Abnormal corticospinal tract decussation	*Central Nervous System*<br>- Intellectual disability, mild (in some patients)<br>- Mirror movements, involuntary, usually of the upper limb and hand<br>- Difficulties in fine bimanual activities<br>- Writing fatigability<br>- Abnormal corticospinal tract decussation<br>- Agenesis of the corpus callosum (in some patients)<br>- Absence of hippocampal commissure	*Central Nervous System*<br>- Mirror movements, involuntary, affecting the hand and fingers<br>- Difficulties in fine bimanual activities
MISCELLANEOUS (in 4/4) ▼	- Onset in infancy or early childhood<br>- Disorder usually remains stable over time<br>- Incomplete penetrance (50%)	- Onset in early childhood<br>- Three unrelated families have been reported (last curated December 2018)	- Onset in infancy or early childhood<br>- Disorder usually remains stable over time<br>- Highly variable phenotype<br>- Incomplete penetrance	- Onset in infancy or early childhood<br>- One consanguineous Pakistani family has been reported (last curated October 2014)
MOLECULAR BASIS (in 4/4) ▼	- Caused by mutation in the homolog of S. cerevisiae RAD51 gene (RAD51, 179617.0003)	- Caused by mutation in the netrin 1 gene (NTN1, 601614.0001)	- Caused by mutation in the deleted in colorectal carcinoma gene (DCC, 120470.0001)	- Caused by mutation in the dynein, axonemal, light chain 4 gene (DNAL4, 610565.0001)
