# Supplementary material for: NeuroLab 2.0: An Alternative Storyline Design Approach for Translating a Research-Based Summer Experience into an Advanced STEM+M Curriculum Unit that Supports Three-Dimensional Teaching and Learning in the Classroom
Source: J STEM Outreach. Author manuscript; Available in PMC 2024 Jun 4. (PMC11149925; doi:10.15695/jstem/v7i1.03)
Supplement: S3 [file NIHMS1973000-supplement-S3.pdf]

NeuroLab

# Unit support for NGSS* Performance Expectations

## HS-LS-1-1

Students who demonstrate understanding can:

***Construct an explanation based on evidence for how the structure of DNA determines the structure of proteins which carry out the essential functions of life through systems of specialized cells.***

**Assessment Boundary:** Assessment does not include identification of specific cell or tissue types, whole body systems, specific protein structures and functions, or the biochemistry of protein synthesis.

The performance expectation above was developed using the following elements from *A Framework for K-12 Science Education:*

| Science and Engineering Practices | Disciplinary Core Ideas | Crosscutting Concepts |
|---|---|---|
| **Constructing Explanations and Designing Solutions**<br><br>Constructing explanations and designing solutions in 9–12 builds on K–8 experiences and progresses to explanations and designs that are supported by multiple and independent student-generated sources of evidence consistent with scientific ideas, principles, and theories.<br><br>• Construct an explanation based on valid and reliable evidence obtained from a variety of sources (including students' own investigations, models, theories, simulations, peer review) and the assumption that theories and laws that describe the natural world operate today as they did in the past and will continue to do so in the future. | **LS1.A \| Structure and Function**<br><br>• Systems of specialized cells within organisms help them perform the essential functions of life.<br><br>• All cells contain genetic information in the form of DNA molecules. Genes are regions in the DNA that contain the instructions that code for the formation of proteins, which carry out most of the work of cells. (Note: This Disciplinary Core Idea is also addressed by HS-LS3-1.) | **Structure and Function**<br><br>• Investigating or designing new systems or structures requires a detailed examination of the properties of different materials, the structures of different components, and connections of components to reveal its function and/or solve a problem. |

**[HS-LS-1-1 Continues on next page]**

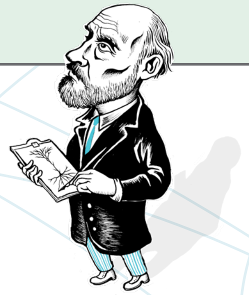

*NGSS is an abbreviated trademark of WestEd.*

## HS-LS-1-1 | Observable features of the student performance by the end of the NeuroLab unit:

### 1. Articulating the explanation of phenomena

Students construct an explanation that includes the idea that regions of DNA called genes determine the structure of proteins, which carry out the essential functions of life through systems of specialized cells

### 2. Evidence

Students identify and describe the evidence to construct their explanation, including that:

a. All cells contain DNA;
b. DNA contains regions that are called genes;
c. The sequence of genes contains instructions that code for proteins; and
d. Groups of specialized cells (tissues) use proteins to carry out functions that are essential to the organism.

Students use a variety of valid and reliable sources for the evidence (e.g., theories, simulations, peer review, students' own investigations).

### 3. Reasoning

Students use reasoning to connect evidence, along with the assumption that theories and laws that describe the natural world operate today as they did in the past and will continue to do so in the future, to construct the explanation. Students describe the following chain of reasoning in their explanation:

a. Because all cells contain DNA, all cells contain genes that can code for the formation of proteins.
b. Body tissues are systems of specialized cells with similar structures and functions, each of whose functions are mainly carried out by the proteins they produce.
c. Proper function of many proteins is necessary for the proper functioning of the cells.
d. Gene sequence affects protein function, which in turn affects the function of body tissues.

## NGSS elements and student performance features supported by the NeuroLab unit:

After observing patients performing a motor task in a clinical setting (the unit kickoff), students are likely to formulate clarifying questions about genetics and the disorder's heritability. We base this assumption on the questions elicited from students during multiple implementation trials of the NeuroLab experience. Accordingly, Lesson 4 (L4) of the NeuroLab unit engages students in a foundational exploration of DNA and the role genes play in specifying the amino acid sequence, structure, and function of proteins used by cells to carry out essential life functions. Understanding of this foundational information is required for students to analyze, interpret, and connect evidence that addresses their initial questions about the role of genes in causing (or contributing to) the movement disorder.

In the next lesson (L5), students implicate genes in the movement disorder by performing a pedigree analysis of family members affected by the disorder. In subsequent lessons (L6 - L9), students discover that the disorder is linked to specific genes (e.g., *DCC* and *NTN1*). They also discover that the proteins encoded by these genes interact and perform specific roles in specialized brain cells (neurons) that enable them to form appropriate functional connections with other specialized neurons involved in voluntary movement (an essential function of life).

To construct their explanation, students will describe and connect evidence obtained from peer-reviewed scientific journal publications and data records from credible databases used by biomedical research scientists and physicians (e.g., NCBI Genome Data Viewer, NCBI ClinVar, Online Mendelian Inheritance in Man, Human Phenotype Ontology, OpenWorm, and Allen Brain Atlas). Students will also describe evidence that they generate using Clustal Omega, EMBOSS Transeq, Simple Modular Architecture Research Tool, and other open-access scientific resources.

The pathway of student exploration, the evidence that students encounter and connect across lessons, and the pedagogical routines adopted by teachers will enable students to demonstrate all three performance features when describing the genetic component/dimension of their explanatory model.

**Note:** As indicated in the first table for this performance expectation (PE), assessment does not include identification of specific cell or tissue types, whole body systems, specific protein structures and functions, or the biochemistry of protein synthesis. However, the explanatory model created by students during the NeuroLab pathway will include basic descriptions of the central nervous system, some of its component cells (e.g., upper and lower motor neurons), specific protein structures and functions (e.g., DCC as a transmembrane receptor displayed at the surface of upper motor neurons and Netrin-1 as a secreted protein that binds DCC to mediate axon pathfinding). Student descriptions of genetic component/dimension of their explanatory model will therefore cross the assessment boundary for this PE.

## HS-LS-1-2

Students who demonstrate understanding can:

***Develop and use a model to illustrate the hierarchical organization of interacting systems that provide specific functions within multicellular organisms.***

**Clarification Statement:** Emphasis is on functions at the organism system level such as nutrient uptake, water delivery, and organism movement in response to neural stimuli. An example of an interacting system could be an artery depending on the proper function of elastic tissue and smooth muscle to regulate and deliver the proper amount of blood within the circulatory system.

**Assessment Boundary:** Assessment does not include interactions and functions at the molecular or chemical reaction level.

The performance expectation above was developed using the following elements from *A Framework for K-12 Science Education:*

| Science and Engineering Practices | Disciplinary Core Ideas | Crosscutting Concepts |
| --- | --- | --- |
| **Developing and Using Models**<br>Modeling in 9–12 builds on K–8 experiences and progresses to using, synthesizing, and developing models to predict and show relationships among variables between systems and their components in the natural and designed worlds.<br>• Develop and use a model based on evidence to illustrate the relationships between systems or between components of a system. | **LS1.A \| Structure and Function**<br>• Multicellular organisms have a hierarchical structural organization, in which any one system is made up of numerous parts and is itself a component of the next level. | **Structure and Function**<br>• Investigating or designing new systems or structures requires a detailed examination of the properties of different materials, the structures of different components, and connections of components to reveal its function and/or solve a problem. |

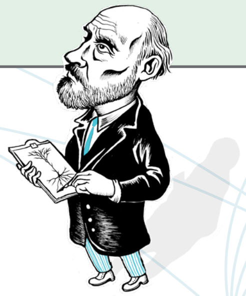

[HS-LS-1-2 Continues on next page]

| HS-LS-1-2 \| Observable features of the student performance by the end of the NeuroLab unit: |
|---|
| **1. Components of the model** |
| Students develop a model in which they identify and describe the relevant parts (e.g., organ system, organs, and their component tissues) and processes (e.g., transport of fluids, motion) of body systems in multicellular organisms. |
| **2. Relationships** |
| In the model, students describe the relationships between components, including:<br>a. The functions of at least two major body systems in terms of contributions to overall function of an organism;<br>b. Ways the functions of two different systems affect one another; and<br>c. A system's function and how that relates both to the system's parts and to the overall function of the organism. |
| **3. Connections** |
| a. Students use the model to illustrate how the interaction between systems provides specific functions in multicellular organisms.<br>b. Students make a distinction between the accuracy of the model and actual body systems and functions it represents. |

**NGSS elements and student performance features supported by the NeuroLab unit:**

This performance expectation (PE) represents the primary student work task of the NeuroLab unit. Throughout the unit, students incrementally construct a model to explain how voluntary movement is produced from interacting systems that operate from the molecular level (e.g., the interaction of proteins involved in guiding the axons of nerve cells to distant target cells during embryonic development) to the whole body systems level (e.g., the interactions among cells within the central nervous system and the muscular system that control voluntary movement).

In Lesson 2 (L2), students explore the hierarchical organization of muscle tissue and examine the process of muscle contraction, which converges on the sliding filament model of muscle contraction. In the second half of this lesson, students explore the basic organization of the brain and spinal cord, and examine the process by which nerve cells within the motor cortex (upper motor neurons) activate muscle cells to produce contraction (via the neuromuscular junction). The interaction of these two systems forms a major focus of student inquiry in subsequent lessons and establishes the foundation for the explanatory model that students construct and revise to explain the anchoring phenomenon (Congenital Mirror Movement Disorder, CMM).

In L3, students analyze and interpret clinical data obtained from CMM patients using electromyelography, transcranial magnetic stimulation, and functional magnetic resonance imaging. By connecting this data with foundational information obtained in L2 and direct observations of the abnormal motor behavior displayed by CMM patients (L1), students discover that the movement disorder is likely to involve a failure of neurons within the corticospinal tract to appropriately activate muscles. This discovery is confirmed in L6 by information contained in data records that students access from authoritative databases (e.g., the Online Mendelian Inheritance in Man and links contained therein).

In subsequent lessons, students discover that genes linked to the movement disorder (*DCC* and *NTN1*) encode proteins that interact during nervous system development to guide the axons of upper (cortical) motor neurons to lower ($\alpha$ spinal cord) neurons on the opposite (contralateral) side of the body axis. They also discover that mutations in these genes disrupt the ability of the corresponding proteins to interact and appropriately guide the axons of upper motor neurons to their target neurons on the contralateral side of the spinal cord.

The pathway of student exploration, the evidence that students encounter and connect across lessons, and the pedagogical routines adopted by teachers are designed to help students demonstrate all three performance features when describing the relevant components/dimensions of their explanatory model.

**Note:** As indicated in the first table for this PE, assessment does not include interactions and functions at the molecular or chemical reaction level. However, the explanatory model created by students during the NeuroLab pathway will include a dimension that explains how a failure of DCC (receptor) to interact with Netrin-1 (ligand) results in a partial misrouting of axons that project from upper (cortical) motor neurons during a key phase of nervous system development (axon pathfinding). Student descriptions of this component/dimension of their explanatory model will therefore cross the assessment boundary for this PE.

## HS-LS-1-4

Students who demonstrate understanding can:

***Use a model to illustrate the role of cellular division (mitosis) and differentiation in producing and maintaining complex organisms.***

**Assessment Boundary:** Assessment does not include specific gene control mechanisms or rote memorization of the steps of mitosis.

The performance expectation above was developed using the following elements from *A Framework for K-12 Science Education:*

| Science and Engineering Practices | Disciplinary Core Ideas | Crosscutting Concepts |
|---|---|---|
| **Developing and Using Models**<br>Modeling in 9–12 builds on K–8 experiences and progresses to using, synthesizing, and developing models to predict and show relationships among variables between systems and their components in the natural and designed worlds.<br>• Develop and use a model based on evidence to illustrate the relationships between systems or between components of a system. | **LS1.B \| Growth and Development of Organisms**<br>• In multicellular organisms individual cells grow and then divide via a process called mitosis, thereby allowing the organism to grow. The organism begins as a single cell (fertilized egg) that divides successively to produce many cells, with each parent cell passing identical genetic material (two variants of each chromosome pair) to both daughter cells. Cellular division and differentiation produce and maintain a complex organism, composed of systems of tissues and organs that work together to meet the needs of the whole organism | **Systems and Model Systems**<br>• Models (e.g., physical, mathematical, computer models) can be used to simulate systems and interactions — including energy, matter, and information flows — within and between systems at different scales. |

[HS-LS-1-4 Continues on next page]

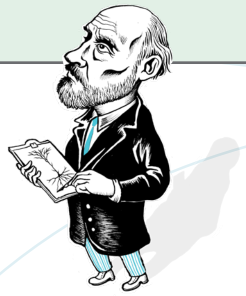

## HS-LS-1-4 | Observable features of the student performance by the end of the NeuroLab unit:

### 1. Components of the model

From the given model, students identify and describe the components of the model relevant for illustrating the role of mitosis and differentiation in producing and maintaining complex organisms, including:

a. Genetic material containing two variants of each chromosome pair, one from each parent;
b. Parent and daughter cells (i.e., inputs and outputs of mitosis); and
c. A multi-cellular organism as a collection of differentiated cells.

### 2. Relationships

Students identify and describe the relationships between components of the given model, including:

a. Daughter cells receive identical genetic information from a parent cell or a fertilized egg.
b. Mitotic cell division produces two genetically identical daughter cells from one parent cell.
c. Differences between different cell types within a multicellular organism are due to gene expression — not different genetic material within that organism.

### 3. Connections

Students use the given model to illustrate that mitotic cell division results in more cells that:

a. Allow growth of the organism;
b. Can then differentiate to create different cell types; and
c. Can replace dead cells to maintain a complex organism.

## NGSS elements and student performance features supported by the NeuroLab unit:

Support materials created for the NeuroLab unit make extensive use of models to support student understanding of fundamental biological processes, including cell division and differentiation. In Lesson 7 (L7), students interpret a variety of models that highlight the role of cell division and differentiation in the development of nervous system regions involved in voluntary human movement. The development of these regions and their component cells forms an important component/dimension of the explanatory model that students construct to explain the movement disorder (anchoring phenomenon).

Students encounter elements of LS1.B at multiple points in the learning trajectory, most notably in L5 and L7. During L7, students explore multimedia resources that illustrate how the division/proliferation of neuronal progenitor cells during embryonic development expands the number of cells in the central nervous system to increase tissue volume and accommodate neuronal diversity. They also explore models that highlight how the exposure of neuronal progenitor cells to secreted proteins affects their differentiation (identity) and dictates the specific roles they will ultimately play in the mature nervous system.

When asked to explain the cellular and developmental dimensions of their explanatory model, students should be capable of identifying and describing each of the relationships and connections outlined in the sections above. Students should also be capable of identifying the nervous system as a collection of differentiated cells and using a model to highlight: 1) the progression of multi-potent neuronal progenitor cells through the major phases of the cell cycle and into mitosis; 2) symmetrical cell division leading to an expansion of progenitors in the central nervous system; 3) migration of progenitors from a proliferative zone into different regions of the developing central nervous system (where they are exposed to varying levels of secreted morphogens); 4) the role that morphogen gradients play in the differentiation of neuronal progenitors; and 5) the role that differentiation plays in the function of neuronal cell types and the connections they form with other neurons.

**Note:** As indicated in the first table for this PE, assessment does not include specific gene control mechanisms. However, students' description of their explanatory model may include a discussion of how specific morphogens (e.g., Shh, BMPs, Wnts) control the differentiation (cell fate specification) of neuronal progenitor cells and how differentiation influences the pathways taken by axons projecting from different subpopulations of neurons in the central nervous system. Student descriptions of these developmental processes would cross the assessment boundary for this PE.

## HS-LS-3-1

Students who demonstrate understanding can:

***Ask questions to clarify relationships about the role of DNA and chromosomes in coding the instructions for characteristic traits passed from parents to offspring.***

**Assessment Boundary:** Assessment does not include the phases of meiosis or the biochemical mechanism of specific steps in the process

The performance expectation above was developed using the following elements from *A Framework for K-12 Science Education:*

| Science and Engineering Practices | Disciplinary Core Ideas | Crosscutting Concepts |
| --- | --- | --- |
| **Asking Questions and Defining Problems**<br>Modeling in 9–12 builds on K–8 experiences and progresses to using, synthesizing, and developing models to predict and show relationships among variables between systems and their components in the natural and designed worlds.<br>• Develop and use a model based on evidence to illustrate the relationships between systems or between components of a system. | **LS1.A \| Structure and Function**<br>• All cells contain genetic information in the form of DNA molecules. Genes are regions in the DNA that contain the instructions that code for the formation of proteins. (secondary) (Note: This Disciplinary Core Idea is also addressed by HS-LS1-1.)<br>**LS3.A \| Inheritance of Traits**<br>• Each chromosome consists of a single very long DNA molecule, and each gene on the chromosome is a particular segment of that DNA. The instructions for forming species' characteristics are carried in DNA. All cells in an organism have the same genetic content, but the genes used (expressed) by the cell may be regulated in different ways. Not all DNA codes for a protein; some segments of DNA are involved in regulatory or structural functions, and some have no as-yet known function. | **Cause and Effect**<br>• Empirical evidence is required to differentiate between cause and correlation and make claims about specific causes and effects. |

[HS-LS-3-1 Continues on next page]

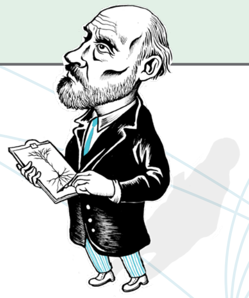

| HS-LS-3-1 \| Observable features of the student performance by the end of the NeuroLab unit: |
|---|
| **1. Addressing phenomena or scientific theories** |
| Students use models of DNA to formulate questions, the answers to which would clarify:<br>a. The cause and effect relationships (including distinguishing between causal and correlational relationships) between DNA, the proteins it codes for, and the resulting traits observed in an organism;<br>b. That the DNA and chromosomes that are used by the cell can be regulated in multiple ways; and<br>c. The relationship between the non-protein coding sections of DNA and their functions (e.g., regulatory functions) in an organism |
| **2. Evaluating empirical testability** |
| Students' questions are empirically testable by scientists. |
| **NGSS elements and student performance features supported by the NeuroLab unit:** |
| Asking questions that clarify relationships relevant to a heritable movement disorder is a prominent feature of the NeuroLab unit. Clarifying questions related to the role of DNA and chromosomes in coding the instructions for characteristic traits passed from parents to offspring become a major driver of inquiry once students discover in Lesson 5 (L5) that the disorder is passed from parents to children in an autosomal dominant fashion and linked to mutations in specific genes *(NTN1* and *DCC)*.<br><br>In subsequent lessons, students formulate and explore questions that clarify how DNA mutations result in the primary motor phenotype (trait) observed in individuals affected by the disorder (bimanual synkinesia/mirror movements). During these lessons, students utilize authoritative databases (e.g., NCBI Genome Data Viewer) and graphical user interfaces (informatics-based models) that show the position of these genes on physical and genetic chromosome maps. Students use NCBI ClinVar and other open-access resources to determine how changes in the nucleotide sequences of these genes can produce changes in the amino acid sequences of the proteins they encode. They then use the Simple Modular Architecture Research Tool and other resources to determine how these changes prevent the proteins from interacting with one another and performing a critical role in nervous system development (e.g., in mediating axon pathfinding).<br><br>Information and data encountered by students throughout the NeuroLab pathway will also enable them clarify an explanation for why these same mutations do not disrupt connections made by other differentiated neurons in the central nervous system (e.g., the pathways taken by axons projecting from other neurons are not affected because they use other navigational systems for axon guidance; the DCC and NTN1 genes are not expressed in these cells because the factors responsible for triggering their expression are either absent or inactive and thus unable to bind to non-coding gene regulatory sequences; etc.). |

## HS-LS-3-2

Students who demonstrate understanding can:

***Make and defend a claim based on evidence that inheritable genetic variations may result from: (1) new genetic combinations through meiosis, (2) viable errors occurring during replication, and/or (3) mutations caused by environmental factors.***

**Clarification Statement:** Emphasis is on using data to support arguments for the way variation occurs

**Assessment Boundary:** Assessment does not include the phases of meiosis or the biochemical mechanism of specific steps in the process.

The performance expectation above was developed using the following elements from *A Framework for K-12 Science Education:*

| Science and Engineering Practices | Disciplinary Core Ideas | Crosscutting Concepts |
|---|---|---|
| **Engaging in Argument from Evidence**<br>Engaging in argument from evidence in 9-12 builds on K-8 experiences and progresses to using appropriate and sufficient evidence and scientific reasoning to defend and critique claims and explanations about the natural and designed world(s). Arguments may also come from current scientific or historical episodes in science.<br>• Make and defend a claim based on evidence about the natural world that reflects scientific knowledge and student-generated evidence. | **LS3.B \| Variation of Traits**<br>• In sexual reproduction, chromosomes can sometimes swap sections during the process of meiosis (cell division), thereby creating new genetic combinations and thus more genetic variation. Although DNA replication is tightly regulated and remarkably accurate, errors do occur and result in mutations, which are also a source of genetic variation. Environmental factors can also cause mutations in genes, and viable mutations are inherited.<br>• Environmental factors also affect expression of traits, and hence affect the probability of occurrences of traits in a population. Thus the variation and distribution of traits observed depends on both genetic and environmental factors. | **Cause and Effect**<br>• Empirical evidence is required to differentiate between cause and correlation and make claims about specific causes and effects. |

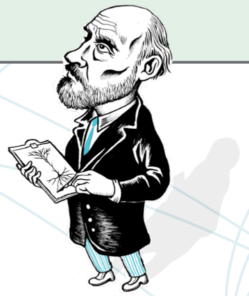

**[HS-LS-3-2 Continues on next page]**

| HS-LS-3-2 \| Observable features of the student performance by the end of the NeuroLab unit: |
|---|
| **1. Developing a claim** |
| Students make a claim that includes the idea that inheritable genetic variations may result from:<br>a. New genetic combinations through meiosis;<br>b. Viable errors occurring during replication; and<br>c. Mutations caused by environmental factors. |
| **2. Identifying scientific evidence** |
| Students identify and describe evidence that supports the claim, including:<br>a. Variations in genetic material naturally result during meiosis when corresponding sections of chromosome pairs exchange places.<br>b. Genetic mutations can occur due to:<br>    a. errors during replication; and/or<br>    b. environmental factors.<br>c. Genetic material is inheritable.<br>Students use scientific knowledge, literature, student-generated data, simulations and/or other sources for evidence. |
| **3. Evaluating and critiquing evidence** |
| Students identify the following strengths and weaknesses of the evidence used to support the claim:<br>a. Types and numbers of sources;<br>b. Sufficiency to make and defend the claim, and to distinguish between causal and correlational relationships; and<br>c. Validity and reliability of the evidence. |
| **4. Reasoning and synthesis** |
| Students use reasoning to describe links between the evidence and claim, such as:<br>a. Genetic mutations produce genetic variations between cells or organisms.<br>b. Genetic variations produced by mutation and meiosis can be inherited.<br>Students use reasoning and valid evidence to describe that new combinations of DNA can arise from several sources, including meiosis, errors during replication, and mutations caused by environmental factors<br>Students defend a claim against counter-claims and critique by evaluating counter-claims and by describing* the connections between the relevant and appropriate evidence and the strongest claim |

**NGSS elements and student performance features supported by the NeuroLab unit:**

Students should be capable of developing aspects of this claim as they approach the midpoint of the NeuroLab unit. At that time, students have explored a number of foundational ideas and concepts in molecular (L4) and medical (L5) genetics that intersect with LS3.B.

Students' ability to meaningfully engage in most aspects of this PE is reliant upon ideas, concepts, and evidence that they encounter across L5, L6, L8, and L9. For instance, students discover through pedigree analysis that the movement disorder (trait) under investigation results from a heritable mutation that is transmitted from parent to offspring in an autosomal dominant fashion (L4). By L5, students identify several genes that are linked to the disorder, two of which play well-established roles in nervous system development (e.g., *DCC* and *NTN1*). In L9, students discover that patients affected by the movement disorder harbor variants of these genes that contain only minor nucleotide changes that are likely to arise from an error in DNA replication or a failure in DNA repair in response to an undetermined source of damage (e.g., insertions, deletions, duplications, and substitutions). In L8, students examine axon trajectories in nematode worms that bear mutations in *DCC* and *NTN1* orthologs; these organisms are the progeny worms that were exposed to a chemical (environmental) mutagen during a large-scale screen for genes involved in movement.

To make and reason through their claim, students may draw upon, evaluate, and connect evidence from a variety of credible sources, including models that they encounter/interpret in multimedia support resources (including those illustrating the process of DNA replication and/or DNA repair that occurs in response to DNA damage), clinical information that appears in semi-fictitious case studies, data figures adapted from peer-reviewed scientific publications, and data reports displayed scientific databases (e.g., Online Mendelian Inheritance in Man and NCBI ClinVar).
