# Supplementary material for: NeuroLab 2.0: An Alternative Storyline Design Approach for Translating a Research-Based Summer Experience into an Advanced STEM+M Curriculum Unit that Supports Three-Dimensional Teaching and Learning in the Classroom
Source: J STEM Outreach. Author manuscript; Available in PMC 2024 Jun 4. (PMC11149925; doi:10.15695/jstem/v7i1.03)
Supplement: S4 [file NIHMS1973000-supplement-S4.pdf]

NeuroLab

Lesson support for NGSS* Performance Expectations and Opportunities for Assessment

Anchoring Phenomenon: Congenital Mirror Movement Disorder (a biomedical phenomenon affecting voluntary human movement)

Lesson	Focus of Student Exploration	3D Assessment Options (formative)
1	Observing the behavior of individuals affected by a rare movement disorder	This lesson presents an opportunity for students to demonstrate that they can:
	During this opening lesson, students will explore the characteristics and roles of scientific models and observe videos of individuals performing a motor task in a clinical setting.<br><br>Teachers will ask students to formulate questions based their observations of the motor behavior and prior knowledge of disorders affecting human movement. Students will then sort their questions into categories that define investigative areas to explore. Teachers will ask then ask students to prioritize which investigative areas should be explored first.<br><br>At the conclusion of the lesson, teachers will instruct students to create an initial explanatory model (v.1) that incorporates their observations and prior knowledge/experience. Students will be provided with multiple opportunities to revise their model based on the discoveries they make in subsequent lessons. The final model (v.6) will be the target of **summative assessment** in L10.	1. **Ask questions** that arise from careful observation of the individuals depicted in the videos to clarify and seek additional information about why they display the observed movements.<br>2. **Develop an initial explanatory model** (v.1) for the movement disorder.<br><br>At the conclusion of this lesson, students will be asked to create an initial explanatory model (v.1) based on: 1) their limited observations of the movements displayed by individuals in the video and 2) their understanding of disorders affecting people with whom they encountered in their lives.<br><br>Many questions elicited from students during unit development and early implementation trials were centered on the muscular system and the central nervous system (refer to Sample Question Categories and Question Library for specific examples). Initial models are likely to include elements of (and interactions between) these two body systems.

Disciplinary Core Ideas	Crosscutting Concepts
**Initial student questions and the categories they form will align with multiple Disciplinary Core Ideas (or elements/pieces of these DCIs), which are most likely to include:**<br><br>LS1.A Structure and Function<br>LS3.A Inheritance of Traits	**Initial student questions and the categories they form will align with multiple Crosscutting Concepts, which are likely to include:**<br><br>• Structure and Function<br>• Systems and Model Systems<br>• Cause and Effect

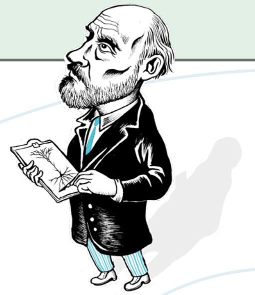

*NGSS is an abbreviated trademark of WestEd.

Anchoring Phenomenon: Congenital Mirror Movement Disorder (a biomedical phenomenon affecting voluntary human movement)		
**Lesson**	**Focus of Student Exploration**	**3D Assessment Options (formative)**
2	Exploring the body systems involved in human movement	This lesson presents an opportunity for students to demonstrate that they can:
	In Lesson 2A (L2A), students will explore the hierarchical organization of muscle tissue and examine the process of muscle contraction, which converges on the sliding filament model of muscle contraction. In the second half of this lesson (L2B), students will explore the basic organization of the brain and spinal cord, and examine the process by which nerve cells within the motor cortex (upper motor neurons) activate muscle cells to produce contraction (via the neuromuscular junction).<br>Refer to Sample Question Categories and Question Library for specific examples of student questions aligned with this investigative area.	1. **Obtain and communicate information** about the basic structure and function of skeletal muscle cells that are presumed to be involved in the movement displayed by individuals shown in the videos.<br>2. **Obtain and communicate information** about the basic structure and function of mature nerve cells that are presumed to be involved in the movement displayed by individuals shown in the videos.<br>3. **Develop and use a model** to illustrate the relationships between the muscular system and the nervous system and how their component parts may interact to produce the movements displayed by individuals depicted in the videos.
**Disciplinary Core Ideas**		**Crosscutting Concepts**
**Student performance demonstrations will incorporate multiple Disciplinary Core Ideas (or elements/pieces of these DCIs), which are most likely to include:**<br>LS1.A Structure and Function		**Student performance demonstrations (and the model they develop) will incorporate aspects of the following Crosscutting Concepts:**<br>• Structure and Function<br>• Systems and Model Systems<br>• Cause and Effect<br>• Scale, Proportion, and Quantity

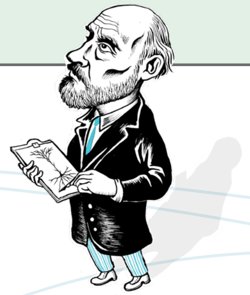

Anchoring Phenomenon: Congenital Mirror Movement Disorder (a biomedical phenomenon affecting voluntary human movement)

Lesson	Focus of Student Exploration	3D Assessment Options (formative)
3	Interpreting the results of diagnostic tests of movement	This lesson presents an opportunity for students to demonstrate that they can:
	In L3A-L3C, students will analyze and interpret clinical data obtained from CMM patients using electromyelography, transcranial magnetic stimulation, and functional magnetic resonance imaging. By connecting this data with foundational information obtained in L2 and direct observations of the abnormal motor behavior displayed by CMM patients (L1), students will discover that the movement disorder is likely to involve a failure of axons within the corticospinal tract to appropriately activate muscles. This possibility is confirmed in L6 by information that students obtain from the Online Mendelian Inheritance in Man database (OMIM).<br><br>Refer to Sample Question Categories and Question Library for specific examples of student questions aligned with this investigative area.	1. **Analyze and interpret data** obtained from peer-reviewed clinical studies to under stand how muscles are activated in people affected by the movement disorder.<br>2. **Develop an interim explanatory model** (**v.2**) for the movement disorder based on a synthesis of the information and evidence encountered in L1 – L3.<br><br>At the conclusion of this lesson, students will be asked to revise their initial explanatory model by incorporating and connecting discoveries made in L1 – L3. The scoring rubric for the revised model (v.2) contains a reference table listing each major discovery and the information/evidence students encountered to make the discovery. Teachers will use the rubric to formatively assess the development of student models and student understand-ing of the concepts, ideas, and evidence used to construct them. During discussions of the model and its components or dimensions (e.g., behavioral, genetic, neurophysiological), teachers may also assess student progress toward meeting the lesson-level performance expectations listed for L1 – L3 and the ability of students to **engage in argument from evidence** when defending or critiquing models.

Disciplinary Core Ideas

Student performance demonstrations (and the model they develop) will incorporate multiple Disciplinary Core Ideas (or elements/pieces of these DCIs), which are most likely to include:

LS1.A Structure and Function

LS1.B Growth and Development of Organisms

LS3.A Inheritance of Traits

Crosscutting Concepts

Student performance demonstrations (and the model they develop) will incorporate aspects of the following Crosscutting Concepts:

- Structure and Function
- Systems and Model Systems
- Cause and Effect
- Scale, Proportion, and Quantity

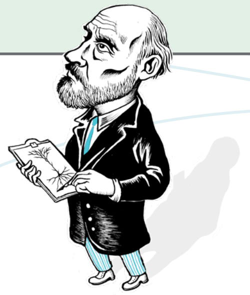

Anchoring Phenomenon: Congenital Mirror Movement Disorder (a biomedical phenomenon affecting voluntary human movement)

Lesson	Focus of Student Exploration	3D Assessment Options (formative)
4	Exploring molecular genetics	This lesson presents an opportunity for students to demonstrate that they can:
	In L1 students are likely to formulate questions about the disorder's heritability or the involvement of genes in its expression. This lesson engages students in a foundational exploration of chromosomes, DNA, and the role genes play in specifying the amino acid sequence, structure, and function of proteins used by cells to carry out essential life functions. Understanding of this foundational information is required for students to analyze, interpret, and connect evidence that links specific gene mutations to the movement disorder.<br><br>Refer to Sample Question Categories and Question Library for specific examples of student questions aligned with this investigative area.	1. **Obtain and communicate information** about the role of genes in directing the synthesis of proteins in component cells of the body systems that students have implicated in the movement disorder (e.g., the muscular system and the central nervous system).

Disciplinary Core Ideas	Crosscutting Concepts
**Student performance demonstrations will incorporate multiple Disciplinary Core Ideas (or elements/pieces of these DCIs), which are most likely to include:**<br><br>LS1.A Structure and Function<br>LS3.A Inheritance of Traits	**Student performance demonstrations (and the model they develop) will incorporate aspects of the following Crosscutting Concepts:**<br><br>• Structure and Function<br>• Systems and Model Systems<br>• Cause and Effect

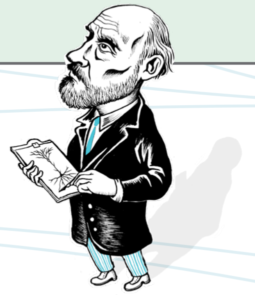

Anchoring Phenomenon: Congenital Mirror Movement Disorder (a biomedical phenomenon affecting voluntary human movement)

Lesson	Focus of Student Exploration	3D Assessment Options (formative)
5	Exploring medical genetics	This lesson presents an opportunity for students to demonstrate that they can:

In this lesson, students will explore select examples of dominant and recessive gene alleles and their role in the expression of a particular phenotype (trait). They will also explore the use of Punnet Squares to predict the probability that offspring will inherit a phenotype (trait) from their parents, and the use of pedigree charts to show actual patterns of inheritance through multiple generations of a family.

During this lesson students will also carry out an investigation to determine if the movement disorder (trait) under investigation results from a heritable mutation and to determine the pattern of inheritance (e.g., autosomal dominant).

In the final part of this lesson, students will use a web-based application within the Human Phenotype Ontology database to diagnose the movement disorder. To perform the diagnosis, students indicate the disorder's pattern of inheritance (obtained through pedigree analysis) and enter clinical features (symptoms) displayed by affected patients (students obtain this information from case reports).

The diagnosis report generated by the Phenomizer app not only includes the name of the disorder, but the name of genes linked to the disorder. The role of these genes in the movement disorder will be the focus of student exploration in subsequent lessons.

Refer to Sample Question Categories and Question Library for specific examples of student questions aligned with this investigative area.

1. **Plan and carry out an investigation** to determine if the movement phenotype is passed down from one generation to the next. Students will use information contained in case studies to carry out their investigation, which involves the creation of a pedigree chart.
2. **Use mathematics and computational thinking** to explain the involvement of DNA, chromosomes, and genes in the transmission of the motor phenotype (bimanual synkinesia) from parents to a proportion of their offspring.
3. **Analyze and interpret data** obtained from pedigree analysis to determine the phenotype's mode (pattern) of inheritance.
4. **Obtain and communicate information** about the identity of genes linked to the movement disorder.
5. **Use mathematics and computational thinking** to explain how the Phenomizer application generates and ranks diagnoses with a controlled vocabulary.
6. **Develop an interim explanatory model** (v.3) for the movement disorder based on a synthesis of the information and evidence encountered in L1 – L5.

At the conclusion of this lesson, students will be asked to revise their explanatory model (v.2) by incorporating and connecting discoveries made in L1-L5. The scoring rubric for the revised model (v.3) contains a reference table listing each major discovery and the information/evidence students encountered to make the discovery. Teachers will use the rubric to formatively assess the development of student models and student understanding of the concepts, ideas, and evidence used to construct them. During discussions of the model and its components or dimensions (e.g., behavioral, genetic), teachers may also assess student progress toward meeting the lesson-level performance expectations listed for L1 – L5 and the ability of students to **engage in argument from evidence** when defending or critiquing models.

Disciplinary Core Ideas

Student performance demonstrations will incorporate multiple Disciplinary Core Ideas (or elements/pieces of these DCIs), which are most likely to include:

LS1.A Structure and Function

LS3.A Inheritance of Traits

Crosscutting Concepts

Student performance demonstrations (and the model they develop) will incorporate aspects of the following Crosscutting Concepts:

- Structure and Function
- Systems and Model Systems
- Cause and Effect
- Scale, Proportion, and Quantity

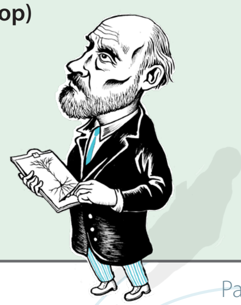

Anchoring Phenomenon: Congenital Mirror Movement Disorder (a biomedical phenomenon affecting voluntary human movement)

Lesson	Focus of Student Exploration	3D Assessment Options (formative)
6	Identifying genes and gene products linked to the movement disorder	This lesson presents an opportunity for students to demonstrate that they can:

In this lesson, students will use the Online Medelian Inheritance in Man database to identify four genes linked to the movement disorder. By analyzing data records contained in the database, students discover that the proteins encoded by two of these genes (*NTN1* and *DCC*) interact during a key phase of nervous system development (axon pathfinding). The information that students obtain from OMIM data records also builds upon a key discovery made in L3 (i.e., the movement disorder results from a failure of axons within the corticospinal tract to appropriately activate hand and limb muscles).

1. **Obtain and communicate information** about the identity of genes linked to the movement disorder and the function of the corresponding proteins during nervous system development.
2. **Ask questions** to clarify how gene (DNA) mutations in humans can result in a failure of upper (cortical) motor neurons to form appropriate connections with other specialized cells that control movement.
3. **Develop an interim explanatory model** (**v.4**) to explain how the Phenomizer application generates and ranks diagnoses with a controlled vocabulary.

At the conclusion of this lesson, students will be asked to revise their emerging explanatory model by incorporating and connecting discoveries made in L1-L6. The scoring rubric for the revised model (v.4) contains a reference table listing each major discovery and the information/evidence students encountered to make the discovery. Teachers will use the rubric to formatively assess the development of student models and student understanding of the concepts, ideas, and evidence used to construct them. During discussions of the model and its components or dimensions (e.g., behavioral, genetic, neurophysiological, developmental), teachers may also assess student progress toward meeting the lesson-level performance expectations listed for L1 – L6 and the ability of students to **engage in argument from evidence** when defending or critiquing models.

Disciplinary Core Ideas

Student performance demonstrations (and the model they develop) will incorporate multiple Disciplinary Core Ideas (or elements/pieces of these DCIs), which are most likely to include:

LS1.A Structure and Function

LS1.B Growth and Development of Organisms

LS3.A Inheritance of Traits

Crosscutting Concepts

Student performance demonstrations (and the model they develop) will incorporate aspects of the following Crosscutting Concepts:

- Structure and Function
- Systems and Model Systems
- Cause and Effect

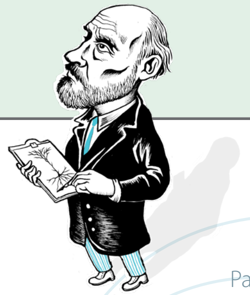

Anchoring Phenomenon: Congenital Mirror Movement Disorder (a biomedical phenomenon affecting voluntary human movement)		
**Lesson**	**Focus of Student Exploration**	**3D Assessment Options (formative)**
**7**	Exploring central nervous system (CNS) development	This lesson presents an opportunity for students to demonstrate that they can:
	In L6, students identified two genes (*NTN1* and *DCC*) linked to the movement disorder. They also determined that the corresponding proteins play important roles in axon pathfinding, a key phase of nervous system development. In L7A, students will explore models that highlight the preceding phases of nervous system development: 1) neurogenesis and neuronal migration and 2) cell differentiation and fate specification.<br><br>In L7B, students will explore axon pathfinding, the process by which differentiated neurons locate target cells with which they will ultimately establish functional connections. As part of this exploration, students will examine the cytoskeletal architecture of the axon and growth cone, both of which display cell surface receptors that are capable of recognizing secreted navigational cues distributed within the developing nervous system (along the pathways taken by pathfinding axons).<br><br>Refer to Sample Question Categories and Question Library for specific examples of student questions aligned with this investigative area.	1. **Obtain and communicate information** about the division/proliferation of neuronal progenitor cells during embryonic development and how this process expands the number of cells in the central nervous system to increase tissue volume and accommodate neuronal diversity.<br>2. **Obtain and communicate information** about the exposure of neuronal progenitor cells to gradients of secreted proteins and how this phenomenon affects their identity (differentiation) and dictates the specific roles they will ultimately play in the mature nervous system.<br>3. **Obtain and communicate information** about how molecular navigation cues guide the axons of differentiated neurons along pathways that lead them to target cells with which they will form functional connections.<br>4. **Ask questions** to clarify how gene (DNA) mutations in humans can result in a failure of upper (cortical) motor neurons to form appropriate connections with other specialized cells that control movement.

Disciplinary Core Ideas	Crosscutting Concepts
**Student performance demonstrations (and the model they develop) will incorporate multiple Disciplinary Core Ideas (or elements/pieces of these DCIs), which are most likely to include:**<br><br>LS1.A Structure and Function<br>LS1.B Growth and Development of Organisms<br>LS3.A Inheritance of Traits	**Student performance demonstrations (and the model they develop) will incorporate aspects of the following Crosscutting Concepts:**<br><br>• Structure and Function<br>• Systems and Model Systems<br>• Cause and Effect

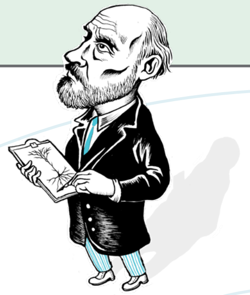

Anchoring Phenomenon: Congenital Mirror Movement Disorder (a biomedical phenomenon affecting voluntary human movement)

Lesson	Focus of Student Exploration	3D Assessment Options (formative)
8	Examining the role of genes in CNS development (model organisms and systems)	This lesson presents an opportunity for students to demonstrate that they can:

In L8A, students use the OpenWorm 3D modeling platform to examine and characterize the trajectories of select C. elegans neurons and make general comparisons to the pathway taken by the axons of upper (cortical) motor neurons that form the corticospinal tract . Students then examine the impacts of Unc-6/*NTN1* and Unc-40/*DCC* mutations on the pathways taken by axons that cross either the dorsal or ventral midline of the C. elegans body axis.

In L8B, students turn their focus to a vertebrate system and examine the trajectories of hindbrain neurons in mice harboring different mutations in the *NTN1* gene.

Refer to Sample Question Categories and Question Library for specific examples of student questions aligned with this investigative area.

1. **Analyze and interpret data** obtained from *C. elegans* and mouse mutants to determine the role played by *NTN1*/Unc-6 and *DCC*/Unc-40 in guiding neurons along the appropriate pathways.
2. **Ask questions** to clarify how gene (DNA) mutations in humans can result in a failure of upper (cortical) motor neurons to form appropriate connections with other specialized cells that control movement.
3. **Obtain and communicate information** about how molecular navigation cues guide the axons of differentiated neurons along pathways that lead them to target cells with which they will form functional connections.
4. **Develop an interim explanatory model** (**v.5**) for the movement disorder based on a synthesis of the information and evidence encountered in L1 – L8.

At the conclusion of this lesson, students will be asked to revise their emerging explanatory model by incorporating and connecting discoveries made in L1-L8. The scoring rubric for the revised model (v.5) contains a reference table listing each major discovery and the information/evidence students encountered to make the discovery. Teachers will use the rubric to formatively assess the development of student models and student understanding of the concepts, ideas, and evidence used to construct them. During discussions of the model and its components or dimensions (e.g., behavioral, genetic, neurophysiological, developmental, cellular), teachers may also assess student progress toward meeting the lesson-level performance expectations listed for L1 – L8 and the ability of students to **engage in argument from evidence** when defending or critiquing models.

Disciplinary Core Ideas

Student performance demonstrations (and the model they develop) will incorporate multiple Disciplinary Core Ideas (or elements/pieces of these DCIs), which are most likely to include:

LS1.A Structure and Function

LS1.B Growth and Development of Organisms

LS3.A Inheritance of Traits

Crosscutting Concepts

Student performance demonstrations (and the model they develop) will incorporate aspects of the following Crosscutting Concepts:

- Structure and Function
- Systems and Model Systems
- Cause and Effect

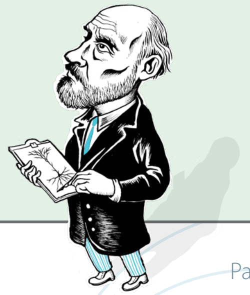

Anchoring Phenomenon: Congenital Mirror Movement Disorder (a biomedical phenomenon affecting voluntary human movement)

Lesson	Focus of Student Exploration	3D Assessment Options (formative)
9	Examining the role of genes in CNS development (human studies)	This lesson presents an opportunity for students to demonstrate that they can:

In the previous lesson, students explored the trajectories of axons in model organisms (*C. elegans* and mouse) bearing mutations in either the *NTN1* or *DCC* gene. In both organisms, mutations in either of these genes resulted in a failure of axons to cross the midline and project on the opposite (contralateral) side of the body axis. In L9A, students will explore diffusion tensor imaging (DTI) data obtained from normal human subjects and individuals affected by the movement disorder. Through this exploration, students discover that mutations in either the *NTN1* or *DCC* genes result in a partial failure of axons within the corticospinal tract to cross the midline at the level of the hindbrain (abnormal corticospinal tract decussation).

In L9B and L9C, students will use online informatics tools and databases, most notably NCBI ClinVar, to examine the consequences of *NTN1* and *DCC* gene variations (mutations) on protein structure and function. Students will also use Simple Modular Architecture Research Tool to explore how mutations impair the ability of Netrin-1 and DCC proteins to interact with one another and perform a role in guiding upper (cortical) motor neurons across the hindbrain midline.

Refer to Sample Question Categories and Question Library for specific examples of student questions aligned with this investigative area.

1. **Analyze and interpret data** obtained from C. elegans and mouse mutants to determine the role played by *NTN1*/Unc-6 and *DCC*/Unc-40 in guiding neurons along the appropriate pathways.
2. **Plan and carry out an investigation** to determine how known mutations in *NTN1* and *DCC* produce changes in the amino acid sequence and structure of the proteins they encode.
3. **Obtain and communicate information** about the nucleotide changes in *NTN1* and *DCC* variants and how these changes affect protein structure and function.
4. **Obtain and communicate information** about the amino acid changes that result from *NTN1* and *DCC* mutations and how these changes affect the ability of the corresponding proteins to interact with one another and perform their functions during axon pathfinding.
5. **Obtain and evaluate scientific information to summarize complex evidence** from multiple organisms to explain how mutations in *NTN1* and *DCC* result in abnormal corticospinal tract decussation in humans affected by the movement disorder.
6. **Ask questions** to clarify where Netrin-1 and DCC proteins are expressed to perform a role in the formation of the corticospinal tract.

Disciplinary Core Ideas

Student performance demonstrations (and the model they develop) will incorporate multiple Disciplinary Core Ideas (or elements/pieces of these DCIs), which are most likely to include:

LS1.A Structure and Function

LS1.B Growth and Development of Organisms

LS3.A Inheritance of Traits

LS3.B Variation of Traits

Crosscutting Concepts

Student performance demonstrations (and the model they develop) will incorporate aspects of the following Crosscutting Concepts:

- Structure and Function
- Systems and Model Systems
- Scale and Proportion
- Cause and Effect

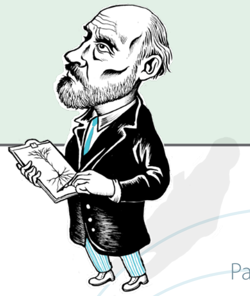

Anchoring Phenomenon: Congenital Mirror Movement Disorder (a biomedical phenomenon affecting voluntary human movement)		
**Lesson**	**Focus of Student Exploration**	**3D Assessment Options (formative)**
10	Completing an explanatory model for the movement disorder	This lesson presents an opportunity for students to demonstrate that they can:
	By this point in the NeuroLab trajectory, students will have implicated defects in the DCC receptor or the Netrin-1 guidance cue as the primary cause of the movement disorder. In this first half of this concluding lesson, students will use the Brain Explorer 2 application and the Allen Developing Mouse Brain Atlas to confirm that the distribution of *NTN1* and *DCC* mRNA in the developing mouse embryo is consistent with the relevant components of their emerging explanatory model.<br>In the second half of this lesson, students will receive guidance on evaluating their model for the presence of different components.	1. **Obtain and communicate information** about the spatial location or cellular localization of Netrin-1 and DCC proteins.<br>2. **Develop an explanatory model** (v.6) for the movement disorder based on the information and evidence encountered in L1 – L10.<br>The scoring rubric for the revised model (v.6) contains a reference table listing each major discovery and the information/evidence students encountered to make the discovery. Teachers will use the rubric for summative assessment of student models and their components. The most comprehensive models will integrate the following components or dimensions: behavioral, neuroanatomical, neurophysiological, molecular genetic, developmental, cellular, and intermolecular.
**Summative assessment** is tied to this concluding lesson and the final explanatory model (v.6) that students construct. Refer to the Unit support for *NGSS Performance Expectations* document for NGSS elements and student performance features supported by the NeuroLab unit.		

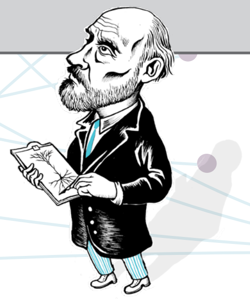

NeuroLab is hosted by Coastal Marine Biolabs and supported by an award from the National Institute of General Medical Sciences, the National Institutes of Health (NIH). The content is solely the responsibility of the authors and does not necessarily represent the official views of the NIH.

SEPA SCIENCE EDUCATION PARTNERSHIP AWARD
Supported by the National Institutes of Health
